# A foot in the door? The impact of a real-world deliberative process on citizens’ attitudes toward deliberative mini-publics (DMPs)

**DOI:** 10.1057/s41599-026-08654-2

**Published:** 2026-08-11

**Authors:** Emilien Paulis, Davide Vittori, Jean-Benoit Pilet, Lisa Verhasselt, Sebastien Rojon, Raphaël Kies

**Affiliations:** 1 University of Luxembourg, Esch-sur-Alzette, Luxembourg; 2 Université Libre de Bruxelles, Brussels, Belgium

**Keywords:** Politics and international relations, Psychology, Sociology

## Abstract

This study examines whether exposure to a deliberative mini-public (DMP) shapes citizens’ attitudes toward this specific form of democratic innovation. Using panel surveys conducted before and after Luxembourg’s 2022 Climate Assembly (Klima-Biergerrot, KBR), we compare three groups: participants (direct exposure), informed non-participants (indirect exposure), and uninformed non-participants (no exposure). Our causal mediation analyses reveal a “foot-in-the-door” effect: when political authorities organize a national DMP, it boosts public support for future DMPs and increases willingness to participate. This effect is powerful when coupled with a positive evaluation of the outcomes and, to a lesser extent, of the process itself. While participants show the greatest attitudinal shift, informed non-participants also exhibit a weaker but similar trend. Overall, organizing a national DMP helps familiarize citizens with deliberative practices and strengthens their legitimacy, thereby contributing to the “deliberative wave” becoming eventually a lasting democratic trend rather than a short-lived experiment.

## Introduction

Over the past two decades, Western democracies have experienced a “deliberative wave” (OECD, 2020), characterized by the increasing use of deliberative mini-publics (DMPs) (Paulis et al., 2021). These forums, composed of randomly selected citizens, deliberate on policy issues to formulate recommendations or, in some cases, make binding decisions (Curato and Farell, 2021). Some policymakers and scholars advocate for further expanding DMPs, viewing them as potential drivers of systemic change that could strengthen or even transform representative democracy (Elstub and Escobar, 2019; Fishkin, 2018; Felicetti, 2021; Geissel and Newton, 2012; Setälä, 2017; Smith, 2009). The OECD (2020:3) has suggested that embedding deliberation into policymaking could mark the beginning of a broader institutional transformation.

However, for such expectations to be realized, broad public engagement with DMPs is, in theory, necessary. And, so far, empirical studies show that this condition is not yet met. While there is some public support for integrating DMPs into decision-making, it remains limited (Jacquet et al., 2022; Pow, 2023; Pilet et al., 2023) and primarily favors an advisory role rather than a binding influence (Bedock and Pilet, 2023; Goldberg et al., 2024). This stands in contrast to the widespread public support for binding referendums (Schuck and De Vreese, 2015). Furthermore, support for DMPs tends to be concentrated among citizens who are more critical of representative democracy (Goldberg and Bächtiger, 2023; Pilet et al., 2024), and willingness to personally participate in DMPs remains low (Jacquet, 2017). A common explanation for this lack of engagement is citizens’ unfamiliarity with DMPs, how they function, their role in policymaking, and their broader democratic potential (Germann et al., 2024; Goldberg et al., 2020). A proposed solution is to increase public exposure by organizing more DMPs, thereby fostering familiarity and encouraging institutionalization through a “foot-in-the-door” strategy.

Our study empirically examines whether exposure to a real-world DMP increases public engagement with such participatory processes. In doing so, we aim to make two key contributions, which are both theoretical and empirical, to the literature on citizens’ support for DMPs. The first contribution is to assess the impact of exposure to a real-world case of a DMP commissioned by a public authority, and whether it could modify how citizens are ready to engage with DMPs. While there is a solid strand of research on the transformative effect on DMP participants (Jacquet and Van Der Does, 2021, Theuwis et al., 2024), less is known about how exposure affects non-participating citizens. Some experimental studies suggest that indirect information about DMPs can generate positive attitudes of the broader public (Boulianne, 2018; Germann et al., 2024; Knobloch et al., 2020), but it remains unclear whether these findings hold in real-world settings where DMPs are organized with clear prerogatives by important political authorities like the national parliament or the government. Moreover, the paper advances existing work by moving beyond evaluations of specific deliberative processes or their immediate outcomes to theorize public attitudes toward DMPs as a generalized democratic instrument. While prior research has predominantly focused on perceptions of procedural quality, legitimacy, or acceptance of recommendations in concrete cases, our contribution lies in conceptualizing and empirically assessing not only support for DMPs, but also the willingness to participate, and the potential support for their binding power as distinct but related dimensions of democratic preferences. We believe these two aspects might be key conditions for their sustainable integration into contemporary democratic systems.

The second contribution of this study is to identify and test mechanisms explaining why exposure to a DMP would generate positive views among citizens about DMPs in general. Building on, and contributing to, research on participatory institutions and democratic legitimacy (Arnesen, 2017; Esaiasson et al., 2019; Werner and Marien, 2022), we explore two theoretical mechanisms that may drive positive attitudes toward DMPs. The first is process-based legitimacy, based on the nature of the process and its intrinsic qualities – how it is organized and who participates (Font and Blanco, 2007; Germann, 2025; Jäske, 2019; Paulis et al., 2024b). When exposed to a DMP, citizens may thus become more supportive of DMPs the more they perceive these processes as fair, inclusive, and well-organized. The second mechanism is outcome-contingency. When evaluating a decision-making process, citizens usually care primarily about the content of the policy recommendations or decisions that are made (Beiser-McGrath et al., 2022; Strebel et al., 2019). Therefore, when exposed to a DMP, citizens may base their evaluation primarily on their evaluation of the policy recommendations rather than the deliberative process itself.

To examine these dynamics, we also present a methodological innovation, which is the third contribution of this article. We use the case of Luxembourg’s 2022 Climate Assembly (Klima Biergerrot – KBR), the country’s first nationally organized DMP, and we took advantage of a panel survey data collected before and after the KBR’s deliberations, covering both participants and a representative sample of the general public. This allows us to distinguish three groups of citizens based on how they were exposed to the KBR: citizens who were never exposed and remained unaware of the KBR throughout the process (no exposure: uninformed non-participants), citizens who became informed about the KBR but did not participate (indirect exposure: informed non-participants), and citizens who directly participated in the KBR (direct exposure: participants). By analyzing how different levels of exposure to a real-world DMP shape attitudes toward DMPs in general, this study provides new insights into the conditions under which DMPs can gain wider public acceptance and legitimacy.

## Theoretical framework

### The impact of exposure to DMPs on participants and informed non-participants

The growing use of mini-publics (OECD, 2020; Paulis et al., 2021) has spurred significant research on this democratic innovation (Jacquet and Van Der Does, 2021; Theuwis et al., 2024). Empirical studies have largely followed two paths. One strand examines the impact of participation in DMPs. The expectation has been that this direct exposure would have a transformative effect on citizens who participate directly, enhancing their political skills, increasing their political interest, and fostering greater civic and political engagement (Smith and Wales, 2000). Additionally, DMPs were hoped to restore political trust and strengthen the legitimacy of political decisions (Dryzek et al., 2019). Over the past decade, scholars have increasingly tested these expectations (Andersen and Hansen, 2007; Boulianne, 2019, Boulianne et al., 2020; Dryzek et al., 2019; Fishkin, 2018; Gastil et al., 2008; Grönlund et al., 2010, 2014; Paulis and Pospieszna, 2024; Sanders, 2012; Strandberg et al., 2021; Volodin, 2019). A meta-review (Van Der Does and Jacquet, 2023) suggests that, in the short term, direct exposure to DMPs does have transformative effects, often increasing participants’ political engagement and knowledge.

The second major strand of DMP research focuses on public attitudes toward DMPs. First, an increasing number of studies have examined public support for expanding the role of DMPs in policymaking and which citizens are most supportive (Jacquet et al., 2022; Pow, 2023; Pilet et al., 2023, 2024; Goldberg and Bächtiger, 2023). This literature also explores the factors shaping support for DMPs (Pow et al., 2020; Van Dijk and Lefevere, 2023; Goldberg et al., 2024). Additionally, a series of (experimental) studies have investigated whether indirect exposure—receiving information about DMPs without participating—affects attitudes toward DMPs, their policy recommendations, and the political authorities that organize them (Boulianne, 2018; Germann et al., 2024; Goldberg et al., 2024; Goovaerts et al.,^ 2025^; Ingham and Levine, 2018; Jacobs and Kaufmann, 2021; Muradova and Suiter, 2022; Már and Gastil, 2023, 2025; Van Der Does and Jacquet, 2023). Findings indicate some significant, though often small, effects.

Surprisingly, few attempts have been made to connect both approaches to real-world DMPs and how the public engages with them. In particular, there has been no direct empirical assessment of the effect of direct exposure (participants) and indirect exposure (informed non-participants), compared to no exposure (uninformed non-participants). Research on the impact of DMPs typically follows separate tracks for participants and non-participants (Van Der Does and Jacquet, 2023). Participants engage directly and extensively with the DMP, interacting with peers and experiencing the process firsthand, making their attitudes more likely to be influenced. In contrast, non-participants have only limited, indirect exposure—typically through media coverage or secondhand information—which likely results in a weaker impact. However, this does not mean that indirect exposure has no effect. Therefore, it is crucial to examine whether there is a difference between citizens who are directly or indirectly exposed to information about DMPs and those who remain completely uninformed about ongoing DMPs in their country, region, or city. Research has shown that even in the case of well-known citizens’ assemblies, the public remains divided between those informed about the mini-public (indirect exposure) and those entirely unaware or uninterested (no exposure) (Carrick and Elstub, 2024; Elstub et al., 2025; Gastil and Knobloch, 2024; Rountree and Curato, 2023; Suiter, 2018).

### The mediating role of perceptions regarding the process and outcomes of DMPs

Our goal in this study is not only to examine whether exposure to a DMP affects attitudes toward DMPs, but also to explore why this might be the case. We aim to understand what factors mediate public support for this form of democratic innovation. To do this, we depart from the literature that has primarily relied on participants’ direct experience with the process to explain attitudinal shift, and instead draw on research on process preferences, which suggests that citizens assess decision-making procedures, and DMPs more specifically, based on two dimensions: (1) the perceived quality of the process through which policy recommendations are made and (2) the content and perceived favorability of these policy recommendations (Arnesen, 2017; Esaiasson et al., 2019; Jäske, 2019; Strebel et al., 2019; Werner and Marien, 2022).

Regarding the first dimension, DMPs can be positively evaluated for several reasons: their inclusiveness, as they allow citizens to participate more directly in policymaking; their (sociodemographic) diversity, which contrasts with the low diversity of elected institutions; the quality of deliberations; and the learning opportunities they provide through expert involvement (Goldberg et al., 2024; Pow et al., 2020; Werner and Marien, 2022). Regarding the second dimension, beyond procedural aspects, research shows that citizens are primarily influenced by policy-related factors. They tend to support decision-making processes when they are positive about the policies that would be—or are—proposed (Arnesen, 2017; Beiser-McGrath et al., 2022; Esaiasson et al., 2019).

Remarkably, past research about the impact of exposure to DMPs has not accounted for these factors. As a second key contribution, this study incorporates the mediating role of citizens’ perceptions of both the process and the outcomes of DMPs. We argue that exposure to a real-world DMP will only foster more positive attitudes toward DMPs in general among individuals who hold positive views on both the process and its outcomes.

## Hypotheses

Building on the theoretical elements discussed in the previous section, we propose testing a series of hypotheses that align with the two key contributions of this study. Our first goal is to examine and compare the effects of direct and indirect exposure to a real-world DMP on citizens’ general attitudes toward DMPs. We expect that exposure—whether direct (as participants) or indirect (as non-participants)—will positively impact citizens’ attitudes toward DMPs, whereas, in contrast, those who receive no specific information about the DMP organized by public authorities are unlikely to experience such changes.

Although the previous subsections have explored how and why direct and indirect exposure to DMPs can transform citizens, they have not precisely defined which attitudes may be affected. As noted, the literature—particularly on DMP participants—sets high expectations for their positive impact, ranging from civic skills and values to political interest, knowledge, and support for democratic institutions and principles (Theuwis et al., 2024). Research on the broader public has focused on how several features affect attitudes the deliberative process (e.g., perceived legitimacy, fairness, and support), its outcomes (e.g., acceptance), as well as toward the commissioning authorities (e.g., trust). In this article, we adopt a broader perspective by examining general attitudes toward, and engagement with, DMPs as a new instrument in contemporary democracies. In line with previous research, we examine support for DMPs, but we also extend the analysis to citizens’ willingness to participate—crucial from the perspective of institutionalization and the integration of DMPs into contemporary democratic systems—as well as to potential support for their binding power. Our goal is to test the “foot-in-the-door” strategy (or “spill over”), which some scholars advocating for deliberative democracy suggest (Van Der Does and Jacquet, 2023). They argue that the growing deliberative wave in modern democracies is just the beginning of a deeper transformation toward a more participatory and deliberative system (Felicetti, 2021; Fishkin, 2018; Setälä, 2017). In this context and acknowledging the need for public endorsement for a genuine deliberative turn, the organization of a DMP in a country is expected to increase support for and engagement with deliberative democracy instruments. In particular, exposure to a real-world DMP should lead to greater demand for replication of DMPs in the future, for granting them more powers, or even to a higher willingness to participate. These three elements will serve as our main dependent variables.

Therefore, the first set of hypotheses (H1-3) breaks down our general expectation about the impact of DMP exposure into three expectations, each related to one of the three key dependent variables that capture different aspects of public support and engagement with DMPs. The first hypothesis posits that exposure to a DMP will increase citizens’ support for organizing similar processes in the future, serving as a proxy for their institutionalization. This hypothesis is grounded in a broad literature highlighting public support for expanding participatory opportunities (Dalton, 2008; Schuck and De Vreese, 2015) and the growing endorsement of DMPs across Europe (Pilet et al., 2023). This trend is often linked to rising dissatisfaction with elected officials and representative democracy (Valgarðsson et al., 2024), prompting citizens to seek greater deliberative engagement and direct involvement in decision-making (Goldberg and Bächtiger, 2023). While some studies indicate that not all citizens favor increased participation (Achen and Bartels, 2016; Hibbing and Theiss-Morse, 2002), the prevailing pattern is a demand for a more inclusive democracy. Moreover, evidence suggests that among DMP participants, positive attitudes toward participation are reinforced after taking part in a mini-public (Paulis and Pospieszna, 2024). This leads us to our first hypothesis.

*H1: Exposure to a real-world DMP increases support for replicating other DMPs*.

At a higher level of attitudinal engagement with DMPs—crucial for sustaining volunteer participation and ensuring diversity—the second hypothesis follows the same logic but focuses on citizens’ willingness to participate in future DMPs. Research suggests that DMPs enhance awareness of civic involvement, often increasing citizens’ general willingness to engage in political action (Grönlund et al., 2010; Myers et al., 2020; Rosenberg, 2007) and, more specifically, in similar deliberative processes (Paulis and Pospieszna, 2024). This self-reinforcing nature of deliberation helps cultivate habits and skills that encourage continued participation in future discursive opportunities (Jacobs and Kauffman, 2019). While this effect is typically observed among DMP participants, it may also extend to non-participants who are merely exposed to the process. Learning about a DMP can spark interest in similar participatory opportunities, potentially reducing reluctance—whether general or in response to an invitation to take part.

*H2: Exposure to a real-world DMP increases willingness to participate in future DMPs*.

Our third hypothesis concerns the most demanding stance on DMPs: support for granting them greater decision-making power. This question ties into broader normative debates on integrating DMPs into the democratic system (Fishkin, 2011; Lafont, 2015). While DMPs are generally viewed as complementary to, rather than substitutes for, representative democracy (Dahl, 1989; Goodin and Dryzek, 2006), Lafont (2015) critiques their potential as decision-making shortcuts, raising concerns about accountability, legitimacy and their disconnect from the broader public. Despite these critiques, citizens may not see such issues as problematic. Instead, they might perceive DMP legitimacy through alternative mechanisms, such as the presence of similar peers and descriptive representation (Pow et al., 2020). If exposure to a DMP enhances political knowledge, skills, and engagement, citizens may come to view its recommendations as more effective than those of elected officials. Additionally, familiarity with binding democratic innovations, such as referendums, could make citizens more open to granting DMPs a similar binding role—an idea sometimes advocated by DMP participants themselves (Paulis et al., 2024a). Based on this, we expect that exposure to a real-world DMP could increase citizens’ support for granting DMPs binding prerogatives.

*H3: Exposure to a real-world DMP increases support for binding DMPs*.

The second key contribution we propose with this study is to examine why exposure to a DMP will generate greater support and engagement towards DMPs. The claim we propose to test is that this positive effect will run through how citizens evaluate the process and the outcomes that will emanate from the DMP. It leads to our second set of hypotheses (H4-H5).

*H4: The positive effect of exposure to a real-world DMP on general attitudes toward DMPs—(a) support for replicating other DMPs, (b) willingness to participate in future DMPs, and (c) support for binding DMPs—is mediated by a positive evaluation of the real-world DMP’s procedural aspects*.

*H5: The positive effect of exposure to a real-world DMP on general attitudes toward DMPs—(a) support for replicating other DMPs, (b) willingness to participate in future DMPs, and (c) support for binding DMPs—is mediated by a positive evaluation of the real-world DMP’s outcomes*.

## Data and method

### Studying a real-world DMP: the Klima Biergerrot (KBR) in Luxembourg

To clearly articulate the key theoretical and empirical contributions this article aims to provide, we developed novel methodological approach to studying the impact of DMPs on citizens. Many existing studies rely on experimental vignettes about hypothetical mini-publics (Boulianne, 2018; Germann et al., 2024; Goldberg and Bächtiger, 2023; Goldberg et al., 2024; Himmelroos and Christensen, 2024; Pilet et al., 2023). We focus instead on real-world exposure to DPMs. The hypothetical nature of such experiments limits external validity, as fictional or small-scale DMPs have little policy impact; in our case, we test the effect of a real-world DMP. While some prior research has analyzed either directly or experimentally actual DMPs, including those organized by public authorities (Elstub et al., 2025; Már and Gastil, 2023; Pow et al., 2020), our study complements this literature by using novel survey data on a nationwide, government-organized DMP, which allows us to assess public reactions over time and to examine both direct and indirect effects of exposure on support for deliberative processes and policy outcomes.

We thus take a different approach rooted in real-life conditions by examining citizens’ reactions to the Klima Biergerrot (KBR), Luxembourg’s first national climate assembly. Announced in 2021 by then-Prime Minister Xavier Bettel, the KBR convened 100 randomly selected citizens (60 full, 40 opt-in) from January to September 2022 to deliberate on climate policy. Their 56 recommendations were submitted to the government and debated in parliament, influencing Luxembourg’s updated National Energy and Climate Plan (NECP) (see Supplementary Appendix A for more details).

**Fig. 1:**
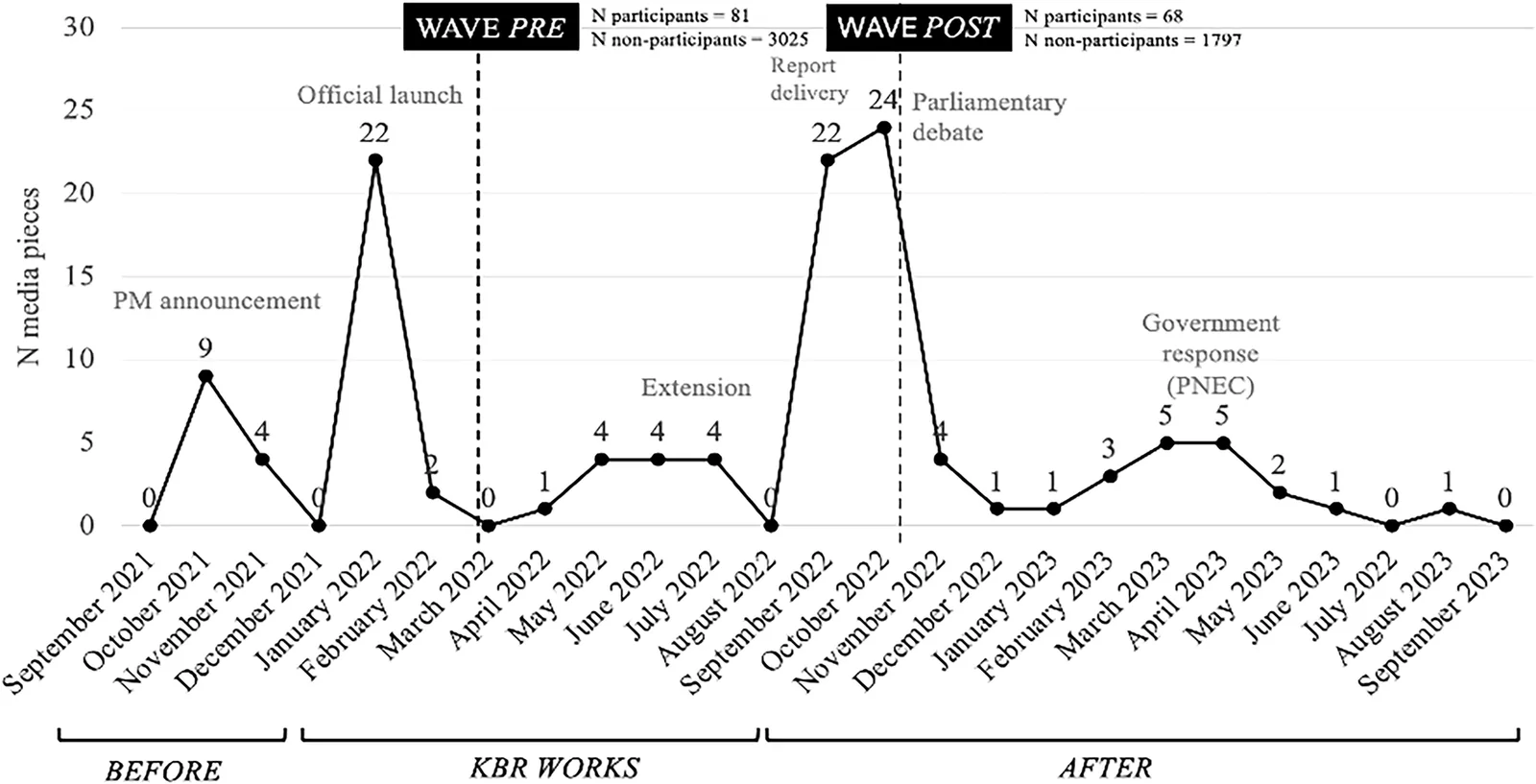
Timeline of the KBR process, media coverage, and survey waves.

The KBR provides a unique case for assessing real-world DMP impact for three reasons. First, unlike experimental settings, it had political significance: the Prime Minister pledged to present its results in parliament, and a dedicated task force reviewed its recommendations, incorporating several into the final NECP. Second, as Luxembourg’s first national DMP, it was free from biases linked to prior deliberative experiences. Third, it received substantial media coverage, particularly at three key moments (Fig. 1): its launch (January 2022), the final report’s submission (September 2022), and the parliamentary debate (October 2022). This sustained attention extended beyond policy proposals to broader discussions on DMPs and their representativeness, fostering public debate and visibility (Paulis et al., 2024c). Our panel survey (see next section) confirms that a significant portion of respondents became aware of the KBR.

### Data

To assess how exposure to a real-world deliberative mini-public (DMP) influences citizens’ general attitudes toward DMPs, our design includes pre- and post-event surveys in order to monitor how the knowledge of the KBR has evolved in the maxi public. We conducted two parallel panel surveys alongside the Klima Biergerrot (KBR) in Luxembourg: one with the DMP participants and another with a representative sample of the national population.

For the DMP participants, we conducted online surveys at the start (pre-DMP) and the end (post-DMP) of the KBR process, which concluded with the final report presentation and parliamentary hearing in November 2022. Participation was voluntary, with informed consent obtained for research and data handling. Of the 100 participants (60 full members and 40 opt-in), 81 completed the pre-DMP survey, and 68 completed the post-DMP survey, yielding a retention rate of 83.9%. Attrition was largely random. The only notable selective pattern concerned age, with younger respondents being significantly more likely to drop out, which slightly increased the overrepresentation of older respondents in the post-wave sample of KBR participants (see Supplementary Appendix B, Supplementary Tables 2B, 3B). This contrasts with the over-representation of young people in the process, at the expense of older participants, especially those aged 65 and over, who were more difficult to recruit in the KBR (Paulis et al., 2024a). In other words, despite being well represented in the process, younger participants were more difficult to capture in the survey and to retain over time.

For the representative Luxembourg sample, we partnered with Ilres, which used quota sampling based on gender, age, education, occupation, region, and nationality, excluding individuals who were selected to become member of the KBR. The initial sample of 3025 respondents completed an online survey in March 2022, shortly after the KBR began (March 8–21). They were re-contacted in November 2022, after the KBR report was presented to Parliament. Of these, 1797 participated in the second wave, yielding a 59.4% retention rate. While the first-wave sample was relatively representative overall—except regarding nationality, which is unsurprising given the difficulty of capturing non-nationals in a Luxembourgish online panel—some selective attrition among older and female respondents led to minor deviations in the post-wave sample (see Supplementary Appendix B, Supplementary Tables 1B, 3B). These were addressed using socio-demographic weights.

The questionnaires for both KBR participants and the general population were identical in wording (see Supplementary Appendix C) and covered four sections: (1) attitudes toward DMPs and the KBR, (2) attitudes toward climate change, (3) political attitudes (interest, trust, efficacy, and knowledge), and (4) socio-demographics. The general population survey included additional questions to assess awareness and understanding of the KBR, allowing respondents to be classified as informed or uninformed non-participants. To accommodate Luxembourg’s multilingual population, the surveys were available in Luxembourgish, French, German, and English. Data quality was ensured through survey-company quality controls – attention, tracking, and speeding (for the general public sample) and an explicit attention check (for the KBR participant sample), in which it was asked to select the figure “10” from a randomized list of five figures (100 percent success rate).

### Operationalization

Our study uses three dependent variables (DVs) from the post-KBR wave to assess citizens’ attitudes toward DMPs. To facilitate the interpretation of effect sizes across models, all dependent variables are scaled from 0 to 1.

1. Support for DMP replication (H1) is measured by agreement with the statement, *“Citizens’ Assemblies like the KBR should be organized on other issues,”* on a scale from 1 (*Fully disagree*) to 5 (*Fully agree*).
2. Willingness to participate in future DMPs (H2) is assessed through the question, *“If selected to join a Citizens’ Assembly or public consultation on climate change or another important issue, how likely would you be to participate?”* Responses range from 0 (*Very unlikely*) to 10 (*Very likely*).
3. Support for binding DMPs (H3) is evaluated by rating the statement, *“Final decisions should be made by a group of randomly selected citizens who deliberate for several days,”* on a scale from 0 (*Bad way of making decisions*) to 10 (*Good way of making decisions*).

Our main independent variable is exposure to the real-world DMP, i.e., the Luxembourg KBR. Among non-participants, we distinguish between those aware and unaware that the DMP was taking place. The survey asked: *“Since the beginning of 2022, Luxembourg organized a national citizens’ assembly, bringing together citizens to deliberate and provide policy recommendations. Before this survey, had you ever heard of this assembly?”* Respondents could answer yes (=1) or no (=0). This self-reporting method is widely used in social science research due to its simplicity, relatively low cost, and, crucially, the assumption that respondents are best placed to assess their own knowledge and experiences. However, we acknowledge an important limitation. With this type of question, respondents may provide answers they believe researchers expect or that portray their knowledge and views in a more favorable light, leading to social desirability bias. That said, the online and confidential nature of the survey helps mitigate this risk, and the questions were formulated in a neutral, non-judgmental manner. To further strengthen our results and to attenuate the social desirability bias, however, we have also replicated our analysis using questions of factual knowledge, instead of self-reporting, to better distinguish between non-participants who were not aware of KBR and non-participants who acquired some knowledge of it (Supplementary Appendix F). The results are robust (see also below).

In terms of coding, respondents who reported to be unaware of the KBR throughout the process were classified as having no exposure (control group). Those aware at any stage (before or after the assembly) are classified as indirect exposure (first treatment group). In the pre wave, 37% of the sample reported knowledge of the KBR, rising to 80% by the post wave. Respondents with inconsistent awareness reports across the two waves (i.e., reporting awareness in the pre but not in the post wave; *N* = 48) were excluded from the analysis. While this proportion may appear high at first glance, it is less surprising when placed in context. Luxembourg is a small country with a dense media environment, and the KBR received substantial public and media coverage, making widespread awareness plausible. Moreover, panel attrition is usually not random: respondents who drop out between waves tend to be less politically interested, whereas those remaining in the post-wave sample are more likely to follow political developments. Nonetheless, a more demanding operationalization that use factual knowledge about the KBR yield substantively similar proportion (see Supplementary Appendix F), suggesting that the over-reporting seems limited. The direct exposure group consists of KBR participants who were directly involved in the process.

To examine why exposure to a DMP influences attitudes, we include two mediators. First, evaluation of the DMP process (H4 – a, b, c) is measured through confidence in the DMP participants, with the statement, *“Participants of citizens’ assemblies like the KBR are informed and skilled enough to contribute to policymaking on climate protection.”* While procedural fairness is a common focus in process evaluations, we chose this parsimonious operationalization anchored in participants’ evaluation because it is a key incentive and bedrock of DMP support (Már and Gastil, 2023; Bedock and Pilet, 2023). DMP support is also linked to social trust (Strandberg et al., 2021). If someone trusts participants’ ability, they are likely to trust the overall process. Additionally, respondents with no exposure to the DMP may have struggled to evaluate the procedural specifics of a process they do not know anything about, and so despite the minimal, generic information that was provided to them at the beginning of the survey block. Asking them to evaluate more specific procedural dimensions—such as representativeness, fairness of selection, or democratic legitimacy—would likely have increased measurement error by requiring judgments about institutional features they did not yet understand. While our survey included other items related to citizens’ assemblies, we chose to rely on a single indicator in the mediation analysis to keep an already complex model tractable and interpretable. We therefore interpret confidence in participants’ competence as a key dimension of process evaluation rather than a comprehensive measure of procedural approval. Second, evaluation of the outcomes (H5 – a, b, c) is assessed through acceptance of the policy recommendations, with the statement, *“I am personally willing to accept the recommendations of citizens’ assemblies like the KBR.”* Both mediators were rated on a 5-point Likert scale from 1 (Fully disagree) to 5 (Fully agree). This is a relatively common, generic way to capture outcome acceptance of deliberative processes in the literature (Paulis et al., 2024b). To conclude, the reason for why our causal analysis include only two mediators is not only theoretical, but also practical, as the package we used in the R software can only handle two mediators at the time.

Our analyses also account for key socio-demographic factors, including gender, age, and education. Given its potential as a confounding factor, we also include political interest. Additionally, we measure climate skepticism, using agreement on a 5-point Likert scale with the statement, *“Claims that human activities are changing the climate are exaggerated.”* Descriptive statistics for all outcome variables, treatment groups, mediators, and controls are detailed in the Appendix.

### Modeling strategy

**Fig. 2:**
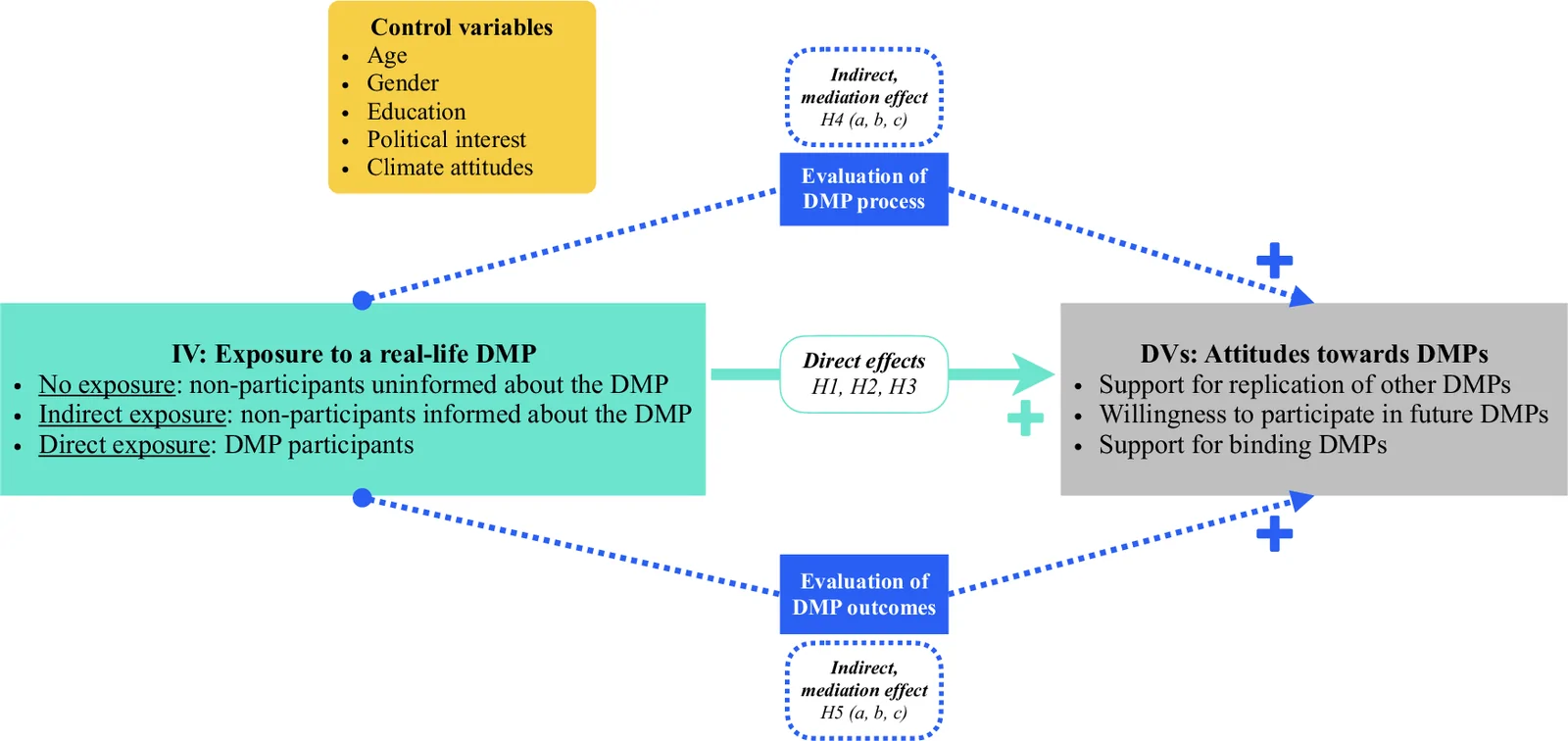
Causal mediation model and hypotheses’ testing.

To test our hypotheses, we use a causal mediation model, which identifies causal pathways presented in Fig. 2. Specifically, we apply the ‘intmed’ package in R to estimate the interventional effects of two mediators, following a counterfactual framework (Chen et al., 2025; Vansteelandt and Daniel, 2017). Our analysis examines both the direct and indirect effects of the main treatment variable—exposure to a real-world DMP (KBR)—through two mediators: evaluation of the DMP process and outcomes. The model controls for gender, age, education, political interest, and climate skepticism to account for potential confounders. Estimation of interventional effects in *intmed* relies on Monte Carlo simulation to approximate counterfactual distributions. Given the presence of categorical covariates with uneven group sizes and the relatively small number of direct DMP participants, we increased the number of Monte Carlo simulations to 3000—three times the default of 1000. This choice improves the numerical stability and precision of the estimated effects by reducing simulation-related Monte Carlo error and ensuring that the results are not sensitive to random variation induced by the simulation procedure. While this does not address limitations related to sample size, it provides greater confidence that the reported estimates reflect the underlying model rather than simulation noise. Full model specifications are available in the Appendices.

Figure 2 illustrates the causal pathways and theoretical expectations. The green path represents the direct effects of the treatment variable (exposure to a real-world DMP) on the three outcome variables, which reflect different general attitudes toward DMPs (H1, H2, H3). The blue path represents indirect effects, where the treatment variable (DMP exposure) influences the three outcome variables through two mediators: DMP process evaluation (H4a, H4b, H4c) and DMP outcome evaluation (H5a, H5b, H5c). Control variables are listed in the top-left box of the figure.

Our analysis proceeds as follows. The main text presents the results of our primary models, where the treatment variable (exposure to the DMP) categorizes respondents into three groups: no exposure, indirect exposure, and direct exposure, using the first group as the baseline. Additionally, we conducted further analyses, detailed in Supplementary Appendices D, E. Supplementary Appendix D compares the two non-participant groups (no exposure vs. indirect exposure) to assess whether our hypotheses about the impact of a real-world DMP hold among non-participants—an understudied group when it comes to attitudinal shift in relation to deliberative processes (Van Der Does and Jacquet, 2023). Supplementary Appendix E contrasts indirect exposure (informed non-participants) vs. direct exposure (participants) to examine whether direct participation in the DMP leads to greater changes than indirect exposure. Any discrepancies with the main model are discussed in the text.

We also conducted a robustness check using an alternative operationalization of the treatment variable, detailed in Supplementary Appendix F. Instead of relying solely on self-reported exposure for non-participants, we assessed their knowledge of the KBR through three questions. Respondents had to identify the correct topic (climate change), participant selection method (civic lottery/sortition), and initiator (the Prime Minister). Non-participants who answered at least one question correctly and claimed to have knowledge of the KBR were classified as “indirect exposure,”, while those who answered none correctly in both the pre- and post-surveys and those who answered one correct, but claimed to have no knowledge of the KBR were categorized as “no exposure.” Supplementary Appendix F provides further details on the descriptive statistics of these knowledge questions, the composition of treatment groups, and the causal mediation analysis. The robustness check results are consistent with those reported in the main text.

## Results

**Fig. 3:**
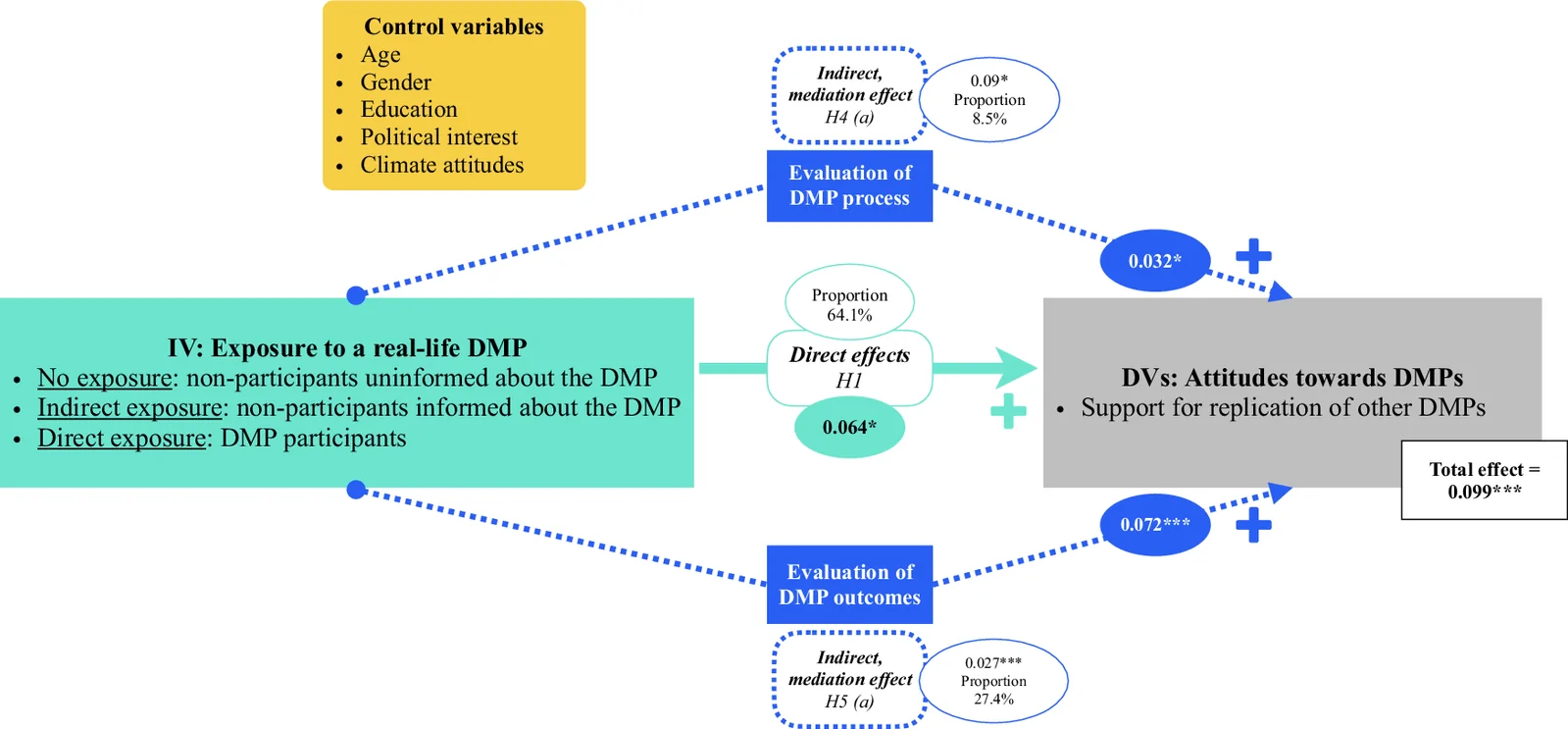
Causal mediation analysis: support for replicating DMPs in the future. ***p ≤ 0.001; **p < 0.01; *p < 0.05.

Figure 3 presents the results of the causal mediation analysis, examining attitudes toward replicating DMPs. Our first hypothesis (H1) proposed that greater exposure to a real-world DMP increases support for replication. The results confirm this expectation, showing a statistically significant, direct, and positive effect of exposure to the KBR on support for organizing other DMPs (ß = 0.064, *p* < 0.05). While the effect size and significance are modest, H1 is supported. However, robustness tests show that this direct effect holds only when comparing direct vs. indirect exposure (see Supplementary Appendix E, Supplementary Table 4E: ß = 0.070, *p* < 0.05) but disappears when comparing indirect vs. no exposure (Supplementary Appendix D, Supplementary Table 4D: ß = 0.019, not significant). This suggests that direct participation in the DMP drives the effect, while merely receiving information is not enough to increase support for replication.

We also hypothesized that the positive effect of DMP exposure would be mediated by evaluations of the process (H4a) and outcomes (H5a). Figure 3 confirms a positive relationship between the treatment variable and the outcome variable through both mediators (blue path): process evaluation (ß = 0.032, *p* ≤ 0.001) and outcome evaluation (ß = 0.072, *p* ≤ 0.001). These findings suggest that support for future DMPs depends on how favorably citizens perceive the recommendations and their confidence in the competence of participants. Among the mediators, the evaluation of DMP outcomes plays a larger role (27.4%) than process evaluation (8.5%). This aligns with previous research, which finds that while process perceptions matter, outcome evaluations are often more influential (Arnesen, 2017; Esaiasson et al., 2019; Jäske, 2019; Sterbel et al., 2019; Werner and Marien, 2022). Still, the direct effect of exposure remains the strongest, accounting for 64.1% of the total effect.

Robustness tests reveal distinct patterns depending on the comparison group. When comparing indirect vs. direct exposure, only the outcome evaluation mediator has a substantial impact (see Supplementary Appendix E, Supplementary Table 4E: ß = 0.060, *p* ≤ 0.001) alongside the direct effect of exposure, while process evaluation is not significant (Supplementary Appendix E, Supplementary Table 4E: ß = 0.006, not significant). When comparing no exposure vs. indirect exposure—focusing on the broader public rather than participants—the direct effect of exposure disappears, and process evaluation becomes significant (see Supplementary Appendix D, Supplementary Table 4D: ß = 0.004, *p* < 0.05). In other words, the direct effect of exposure and the indirect effect of outcome evaluation are primarily driven by the KBR participants (direct exposure), while the indirect effect of process evaluation comes mainly from informed non-participants (indirect exposure).

**Fig. 4:**
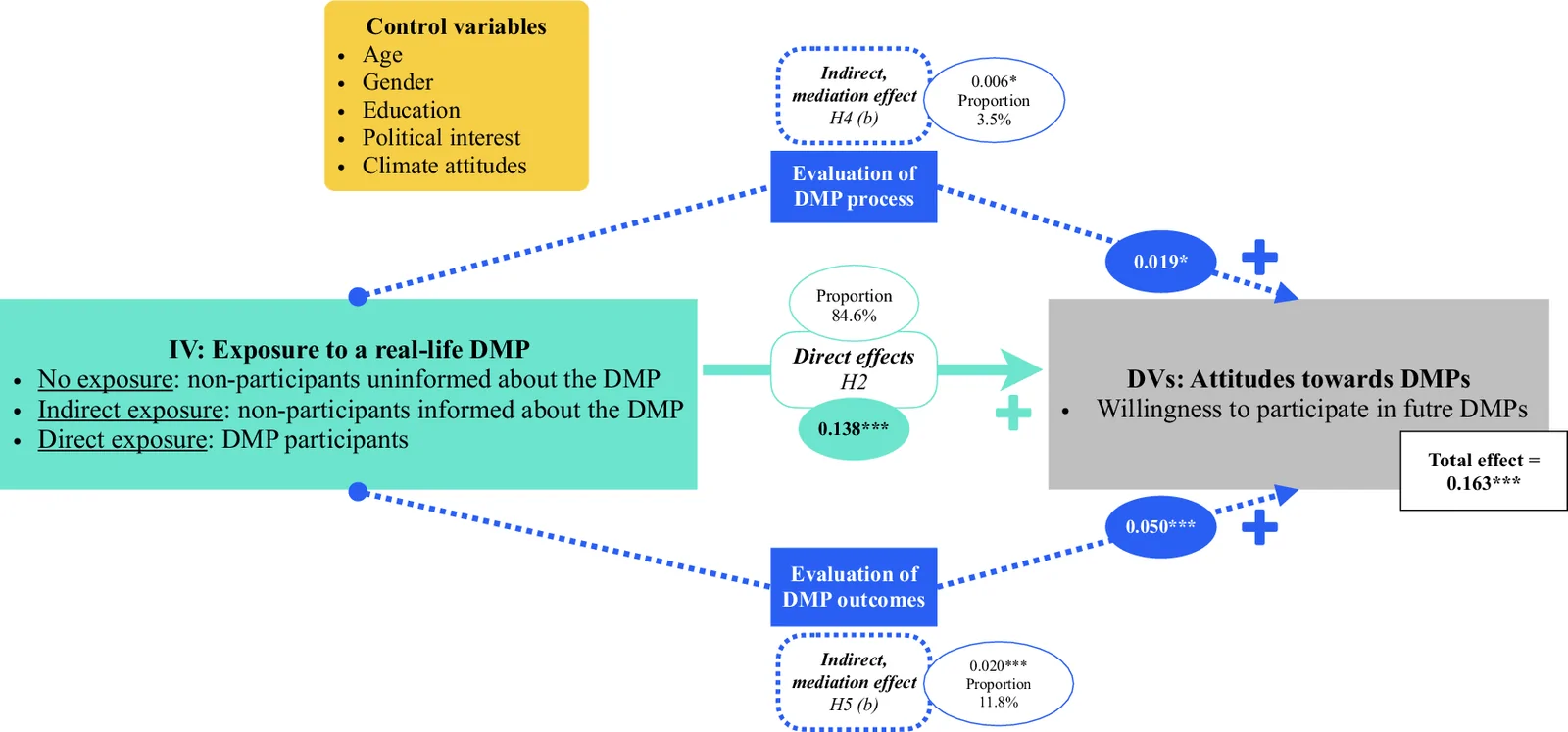
Causal mediation analysis: willingness to participate in future DMPs. ***p ≤ 0.001; **p < 0.01; *p < 0.05.

To test H2, we conducted the same causal mediation analysis, this time using willingness to participate in future DMPs as the dependent variable. As shown in Fig. 4, exposure to the DMP has a direct, statistically significant, and positive effect on citizens’ willingness to participate in future DMPs (ß = 0.138, *p* ≤ 0.001), supporting H2 (green path). Notably, this direct effect remains significant across group comparisons: indirect vs. direct exposure (see Supplementary Appendix E, ß = 0.174, *p* ≤ 0.001), and no exposure vs. indirect exposure (see Supplementary Appendix D, ß = 0.043, *p* < 0.05), with thus a stronger effect in the former case. These findings suggest that real-world DMP exposure can mobilize both participants and informed non-participants. While the effect is strongest for those who directly participated—confirming the “deliberative participation hypothesis” (Grönlund et al., 2010) that suggests that participants are more likely to engage again—it also appears among those who were merely informed about the process.

Examining the indirect effects, our results presented in Fig. 4 support H4b and H5b. There is a positive relationship between the treatment variable and the outcome variable through both mediators (blue path), with the process (ß = 0.019, *p* < 0.05) and outcome evaluations (ß = 0.05, *p* ≤ 0.001) showing significant, positive effects. As with support for organizing additional DMPs, outcome evaluations account for a larger share (11.8%) of the mediation effect than process evaluations (3.5%). However, these indirect effects remain modest, reinforcing that the direct effect of exposure (84.6% of the total effect) is the primary driver of increased willingness to participate. Notably, indirect effects disappear when comparing indirect vs. no exposure (Supplementary Appendix D, Supplementary Table 8D). When comparing direct vs. indirect exposure (Supplementary Appendix E, Supplementary Table 8E), only the outcome evaluation mediator remains significant, though its effect is moderate (ß = 0.037, *p* ≤ 0.001), while the direct effect of exposure is stronger (ß = 0.173, *p* ≤ 0.001). This suggests that the indirect effect found in the main model for outcome evaluations is primarily driven by the KBR participants.

**Fig. 5:**
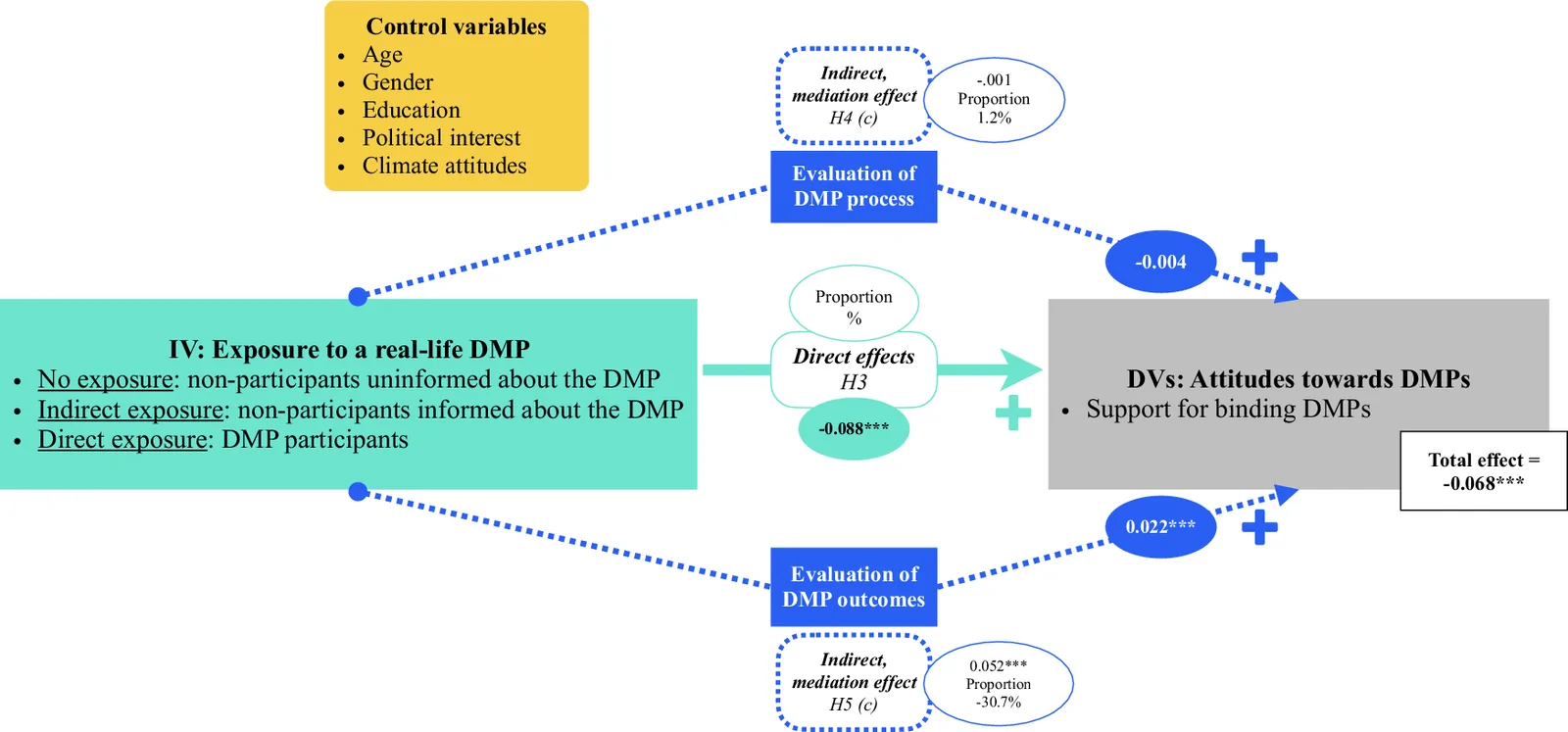
Causal mediation analysis: support for binding DMPs. ***p ≤ 0.001; **p < 0.01; *p < 0.05.

Turning to the third hypothesis (H3) and support for granting decision-making power to DMPs, the results in Fig. 5 diverge from our expectations but provide valuable insights. While exposure to a real-world DMP has a statistically significant, direct effect on support for binding DMPs (green path), the relationship is negative (ß = −0.088, *p* ≤ 0.001). This means that greater exposure coincides with decreased support for granting decision-making power to DMPs. These findings suggest that while exposure increases support for organizing DMPs and willingness to participate, it does not necessarily lead to greater support for expanding their authority. Instead, directly exposed citizens tend to favor keeping DMPs advisory rather than binding. H3 is therefore not supported. This pattern may reflect Luxembourg’s high trust in representative institutions (OECD, 2024), where incentives for democratic reforms are lower. However, it aligns with studies from other countries showing that people generally value DMPs as consultative mechanisms and prefer to limit their authority (Bedock and Pilet, 2023; Goldberg, 2021; Goldberg et al., 2024). This strengthens the external validity of our findings. Furthermore, the negative direct effect holds when comparing direct vs. indirect exposure (see Supplementary Appendix E, Supplementary Table 12E: ß = −0.118, *p* ≤ 0.001). The effect loses significance when comparing indirect vs. no exposure (see Supplementary Appendix D: ß = −0.028, non-significant), suggesting that this shift in attitude is primarily driven by DMP participants. This indicates that direct participation makes individuals less inclined to support binding DMPs. It may also suggest that taking part in the DMP helps individuals better understand the role of participatory mechanisms within the broader political system.

Regarding the two mediators (H4c and H5c), the results provide also a nuanced picture. Process evaluations do not significantly mediate the relationship between DMP exposure and support for binding DMPs (ß = −0.001, non-significant). This mediator accounts for only 1.2% of the total effect, leading to the rejection of H4c. Outcome evaluations, by contrast, show a statistically significant positive mediation effect (ß = 0.022, *p* ≤ 0.001) and account for −30.7% of the total effect. This suggests that while exposure to a DMP generally decreases support for granting decision-making power, a positive assessment of its outcomes can moderate this effect. This means that citizens who view the outcomes favorably are less resistant to expanding DMPs’ authority than those with a more negative perception, confirming H5c. Put differently, despite general skepticism toward binding DMPs, positive evaluations of their outcomes can reduce opposition to expanding their decision-making power. Robustness checks show that the mediating effect of outcome evaluations holds when comparing direct vs. indirect exposure (see Supplementary Appendix E, Table 12E: ß = 0.048, *p* ≤ 0.001), suggesting that participants’ experiences shape their attitudes more than indirect exposure. However, when comparing no exposure vs. indirect exposure, focusing on the broader public only, the mediating effect of both variables disappears (see Supplementary Appendix D, Supplementary Table 12D).

Table 1 summarizes our findings and hypothesis tests. Our results confirm that exposure to a real-world DMP increases support for organizing additional DMPs (H1) and willingness to participate in future ones (H2). This positive effect is mediated by how favorably citizens evaluate both the deliberative process (H4a, H4b) and its outcomes (H5a, H5b), with outcome evaluations playing a stronger role. However, the direct effect of exposure remains the most influential factor. Interestingly, while exposure also shapes views on granting DMPs binding decision-making power, it unexpectedly decreases support for this authority. However, positive evaluations of DMP outcomes moderate this negative effect. Direct participation in deliberation emerges as the main driver behind these effects. As stated in the previous section, we replicated all analyses using an alternative measure of exposure, replacing self-reported awareness of the real-world DMP with a more objective knowledge-based assessment. This adjustment did not change the results in Table 1, further confirming their robustness.

**Table 1:** Summary of the main results.

	Support for DMP replication			DMP participation			Binding DMPs		
	No vs indirect/direct exposure	Indirect vs direct exposure (App. E)	No vs indirect exposure (App. D)	No vs indirect/direct exposure	Indirect vs direct exposure (App. E)	No vs indirect exposure (App. D)	No vs indirect/direct exposure)	Indirect vs direct exposure (App. E)	No vs indirectexposure (App. D)
Direct effect	Yes* (+) **H1**	Yes* (+)	No (+)	Yes*** (+) **H2**	Yes*** (+)	Yes* (+)	Yes** (−) **H3**	Yes*** (−)	No (−)
Indirect: process	Yes* (+) **H4a**	No (+)	Yes* (+)	Yes* (+) **H4b**	No (+)	No (+)	**No (-)** **H4c**	No (−)	No (.)
Indirect: outcomes	Yes** (+) **H5a**	Yes*** (+)	No (+)	Yes*** (+) **H5b**	Yes*** (+)	No (+)	Yes** (+) **H5c**	Yes*** (+)	No (+)

Table 1 summarizes the results of hypotheses tests on the effects of exposure to deliberative mini-publics (DMPs) on support for DMP replication, participation, and binding outcomes. Each cell indicates whether the hypothesis was supported, the significance level (***p ≤ 0.001; **p < 0.01; *p < 0.05), and the direction of the effect in parentheses (+ for positive, – for negative). The table distinguishes between direct effects and indirect effects through process (H4a–H4c) and outcome evaluations (H5a–H5c). Columns compare different types of exposure: “No vs indirect/direct exposure,” “Indirect vs direct exposure (Supplementary Appendix E),” and “No vs indirect exposure (Supplementary Appendix D).” In bold are the results presented in the main text.

Finally, it is important to note that this study examines the impact of a first-ever DMP, which may explain the weaker effects among non-participants who were only informed about its existence. Nevertheless, we find that non-participants exposed to DMP-related information are more willing to participate in future DMPs than those who remained unaware. While overall acceptance remains low (Jacquet, 2017), this suggests that a ‘foot-in-the-door’ strategy could gradually mobilize broader public engagement as more DMPs are organized. Over time, increased familiarity with DMPs may lead to attitudinal spillover, encouraging replication and eventually greater support for granting them binding decision-making power.

## Conclusion

The main goal of this study is to examine whether increasing citizens’ familiarity with DMPs—by exposing them to a real-world DMP in their country—enhances public engagement with this form of democratic innovation. To address this question, we analyze whether the first-ever national-level DMP (a climate assembly called *Klima Biergerrot*) organized by Luxembourg’s government in 2022 affected public attitudes toward DMPs in general, namely whether it increased support for holding more DMPs, willingness to participate in DMPs, and support for granting them binding powers. The underlying argument aligns with what we call the “foot-in-the-door” strategy of DMP advocates in both society and academia. According to this perspective, early deliberative experiences could serve as an initial step toward a broader transformation of democracy, eventually leading to the institutionalization of DMPs. A key expectation is that as citizens gain a better understanding of how DMPs function and their potential benefits, they will perceive them as valuable additions to democratic systems.

Our article makes two key contributions to this debate. First, we examine the effects of exposure to DMPs beyond direct participants. While research suggests that participation in DMPs can foster greater engagement with DMPs, evidence of similar effects among non-participants who receive information about them (i.e., indirect exposure) remains limited (Van Der Does and Jacquet, 2023) though essential for their legitimization. Second, past studies have not thoroughly investigated the mechanisms that explain why exposure to DMPs might increase public support. Our analysis addresses this gap by examining the mediating role of two key factors: citizens’ perceptions of the participants’ capability in deliberative processes and their acceptance of the outcomes produced.

Our results provide key insights into both contributions. First, we find that exposure to a real-world DMP increases support for organizing and participating in future DMPs. While these effects are strongest among direct participants, they also extend to the broader public. This highlights that DMPs are far from anecdotal: if they reach non-participants, they can spark curiosity and encourage broader public participation in the future. However, exposure does not increase support for granting DMPs greater decision-making authority. This aligns with previous studies showing that public support for DMPs is generally higher when they have a consultative rather than binding role (Bedock and Pilet, 2023; Goldberg et al., 2024). It also reflects findings that support for more radical deliberative democracy is primarily found among the most politically disenchanted citizens, who are less likely to revise their views after a government-led mini-public (Bedock and Pilet, 2021; Pilet et al., 2023; van Dijk and Lefevere, 2023).

The second key contribution of our study is in understanding how citizens evaluate DMPs. We confirm that exposure alone is not enough to generate greater public engagement—citizens’ views on both the policy recommendations and the deliberative process itself are crucial. In this regard, our findings support prior research emphasizing the role of process and outcome-related judgments in shaping policy acceptance (Arnesen, 2017; Esaiasson et al., 2019; Jäske, 2019; Sterbel et al., 2019; Werner and Marien, 2022). Furthermore, we found that procedural evaluations and trust in participants’ capabilities matter more for explaining attitudes toward deliberative mini-publics among indirectly informed citizens (especially regarding their support for replication—not significant for participation or binding power), whereas outcome-related evaluations play a larger and consistent role for participants on all outcome variables. Having experienced the deliberative process firsthand, participants’ support is shaped primarily by whether they perceive the outcomes as substantively acceptable and aligned with the preferences they expressed during deliberation. By contrast, indirectly exposed citizens—lacking such firsthand experience about what the outcomes are—probably rely more strongly on procedural cues and trust-based heuristics to assess legitimacy. These insights are essential for those hoping that exposure to real-world DMPs—whether direct or indirect—could pave the way for their institutionalization in contemporary democracies.

Overall, our findings suggest that organizing DMPs is not a shot in the dark. They can play a crucial role in familiarizing citizens with these democratic innovations and strengthening their legitimacy—key factors in ensuring the ‘deliberative wave’ becomes a lasting trend rather than a passing phase. However, our study has limitations that point to areas for further research. First, it captures only short-term shifts in attitudes, not long-term changes, which are central to deliberative democracy theories. Without follow-up data, we cannot assess whether these effects persist beyond the dissolution of the KBR. Second, Luxembourg’s distinctive context—characterized by high levels of education, affluence, and political trust—together with the relatively small number of actual DMP participants, may limit the generalizability of our findings. In addition, the presence of a large foreign resident population, which is unable to vote but may nonetheless be particularly receptive to deliberative mini-publics, introduces an additional layer of contextual specificity (Paulis et al., 2024a). Future research should therefore examine DMPs in larger and more heterogeneous political contexts and, where possible, with larger samples of participants, although such data collection is not always feasible in practice, as DMPs generally are not much bigger than KBR. Third, our analyses include only two mediators, whereas in practice additional mechanisms related to specific characteristics of DMPs may also shape citizens’ attitudes, particularly among participants or individuals with prior knowledge of the process. However, because our research design deliberately includes citizens with no prior knowledge of the mini-public, we cannot meaningfully incorporate mediators that require familiarity with internal DMP features (e.g., deliberation quality or expert involvement). While this constitutes a limitation of the present study, future research focusing exclusively on informed citizens or DMP participants could explicitly examine these additional mediating pathways. Lastly, our study lacks a counterfactual. Media coverage of the KBR was generally positive, particularly when policy recommendations were released (Paulis et al., 2024c). Future studies could analyze DMPs in contexts with varying media narratives and levels of public engagement to provide a more comprehensive understanding of their effects.

## Supplementary information

Supplementary_materials_last

## Data Availability

The data and replication materials supporting the findings of this study are available at https://osf.io/yuqbx/.
