# Supplementary material for: A foot in the door? The impact of a real-world deliberative process on citizens’ attitudes toward deliberative mini-publics (DMPs)
Source: Humanit Soc Sci Commun. 2026 Aug 11;13(1):1885. doi: 10.1057/s41599-026-08654-2 (PMC13695576; doi:10.1057/s41599-026-08654-2)
Supplement: Supplementary file 1 [file 41599_2026_8654_MOESM1_ESM.docx]

**Appendices**

[Appendix A. Description of the KBR: recruitment, organization, an outcomes	1](#_Toc218860204)

[Appendix B. Samples: representativeness and attrition across survey waves	6](#_Toc218860205)

[Appendix C. Questions’ wording, variables’ operationalization and descriptive statistics	9](#_Toc218860206)

[Appendix D – Comparing groups: no exposure versus indirect exposure	12](#_Toc218860207)

[Appendix E – Comparing groups: indirect exposure versus direct exposure	22](#_Toc218860208)

[Appendix F – Actual knowledge of the KBR (instead of self-report): operationalization, distribution and replication	31](#_Toc218860209)

[Appendix G – Causal mediation analysis for Figure 3, Figure 4 and Figure 5: full model specification	42](#_Toc218860210)

# **Appendix A. Description of the KBR: recruitment, organization, an outcomes**

Recruitment

The KBR recruitment was outsourced to the polling institute Ilres. Given the time constrain imposed by the government (Ilreas had only two weeks to form the assembly), Ilres did not fully and only rely on a civic lottery to select the KBR participants. Random selection was combined with self-selection:

- they contacted a sample of 1,500 randomly drawn fixed and mobile numbers (random digit dialing);
- they launched a public call open to volunteers that was relayed by the government and traditional media;
- they shared the same call with people already registered in their existing database or panel (i.e., about 11,000 residing citizens being regularly invited to participate in surveys).

To express their interest, citizens had to fill a small survey on Ilres’ online portal. Interested citizens could also spontaneously contact Ilres and conduct the survey via phone, which was important to reach people less at ease with online technologies. This process generated a pool of 1,100 volunteers. Based on the responses Ilres selected ±100 participants taking into account that when re-contacted to confirm their participation a certain number of people would no longer be interested or have the possibility to take part.

To reflect Luxembourg’s diverse society, Ilres applies stratification quota techniques to select the final participants from the pool of 1,100 volunteers. In their application, citizens provided their sociodemographic information and answered several questions regarding life and climate. Ilres selected thus the final 100 members based on several criteria.

- Age. The minimum age to be recruited was fixed to 16 years old meaning two years less than the official voting age.
- Gender. The KBR was aimed to be a gender-balance citizens’ consultation. This emphasizes the willingness to be more inclusive than in election by mobilizing young adults (below 18 years old) for whom climate is a top policy priority and are generally less politically active.
- Nationality. Ilres took into consideration participants’ nationality. They could have any citizenship, if they spoke at least one of the following languages Luxembourgish, French or English. For the legitimacy of the process, this is essential knowing that Luxembourg is a multilingual society and therefore that deliberation will have to take place in different languages.
- Place of work and residence. Ilres considered whether the selected participants worked in Luxembourg but resided in either Luxembourg, Germany, France, or Belgium. The large share of “cross-border workers” is a peculiarity of the Luxembourg population. Knowing that they are excluded from the electoral process at the national level, but still can greatly be impacted by government (climate) policies, it is crucial for the legitimacy of the process that they are included and have their voices heard.
- Level of formal education. The level of education was also considered to ensure that the selected sample of people was representative of the broader population.
- Citizens holding elected mandates were de facto excluded from the pool of volunteers. By essence, deliberative processes intend to be independent from party politics and related vested interests. They are supposed to prevent office-seeking behaviors of policy makers that would jeopardize the representation of people’s interest.

The demographic criteria (age, gender, level of formal education) resembled to those usually applied in deliberative mini publics (Paulis et al. 2020). The company relied also on less common criteria like language skills, nationality, or place of work and residence, which are nonetheless crucial in the Luxembourg context. No extra efforts or over-sampling was performed to target specific groups in the population that are usually under-represented in politics.

Two further elements were also asked in the survey application and aimed to ensure diversity when selecting the KBR members: satisfaction with life and the importance given to climate protection. The answers given to these two questions were used to ensure that the members were diverse in terms of life and climate attitudes. This is also important because people who are overall less happy and/or climate skeptics may have less incentive to participate in a participatory process, but still need to be represented for their legitimacy.

Organization

The KBR was a citizens’ assembly officially commissioned by the Luxembourg Government via three ministries: the Ministry of State (Prime Minister Xavier Bettel), the Ministry of Environment, Climate and Sustainable Development (Minister Joëlle Welfring), and the Ministry of Energy and Urban Planning (Minister Claude Turmes). KBR participants were tasked with discussing Luxembourg’s current commitments as regards combating climate change, and with developing possible additional measures or proposals. The exact question that was asked to the KBR was the following: Is Luxembourg able and willing to do more to combat climate change? And, if so, how? The KBR was expected to build on the measures already in place to give current climate policy new impetus, drawn directly from Luxembourg society. The KBR was thus assigned to exercise three main functions:

- consider what was already being done in Luxembourg to handle the climate crisis, using the integrated National Energy and Climate Plan as a central focal (NECP);
- consider whether Luxembourg could go further in the fight against climate change; and
- make recommendations about matters in relation to climate change, eventually likely to influence the new version of the NECP.

Aside from the polling institute Ilres which was put in charge of the members’ recruitment of the participants, a consortium of three different firms was appointed for the KBR’s organization. Phase 1 of the KBR took place from February to June 2022. This first phase (and initially planned to be the only phase) included optional online debates, optional in-person study visits, and five compulsory in-person working weekends. To streamline the process and maximize the impact, the KBR organizers structured the consultation in 5 thematic cycles directly related to the 5 policy subthemes of the NECP: agriculture and forestry (weekend 1), renewable energy (weekend 2), sustainable construction (weekend 3), waste management (weekend 4), mobility and transport (weekend 5). Before each working weekend, an optional online debate offered the members (both effective and supplementary) an introduction to the subtheme to be discussed during the weekend by different experts. The expert presentations were followed by a question-and-answer part, allowing members to ask for explanations and additional information, or discuss with experts and between them. These online sessions were recorded and together with presentations of the speakers, shared with all KBR members. Additionally, study or field visits, also optional, were organized before each working weekend. These visits made it possible to meet actors in Luxembourg and to discover their activities, projects, ambitions, and constraints, and exchange directly with them. These visits provided an additional learning experience for the members. Despite their optional nature, attendance was generally high. In the members’ survey, 70% of the respondents reported that they participated in one or several visits before the working weekend(s) that they attended. After the optional online debate and study visit(s), the working weekend took off. These weekends accounted for the main moments of deliberation and conclusion of a thematic cycle. The 60 effective members, or their substitutes, were invited on Saturday afternoon and Sunday morning to explore a specific theme. While Saturday was dedicated to the identification of challenges facing Luxembourg on the topic at hand, on Sunday the members discussed possible solutions. The work was organized between sessions in plenary and in working groups of 12 to 15 members. The KBR members were divided into four language-based working groups, created based on the participants’ own input regarding their language abilities. There was one French, one English and two Luxembourgish groups. Throughout the process, professional moderation was used to create conversations which were inclusive, respectful, to the point, honest and constructive.

During phase 2, taking place from July to August 2022, the members were divided into six groups. Each group focused on a specific topic covered during one of the five working weekends, whilst the sixth group focused on cross-cutting issues. The members were able to register for the thematic working group of their liking and to volunteer as spokesperson/group leaders. Over the summer period, the spokespersons managed their groups largely independently and had four coordinating meetings with the organization and moderation team. Each group received 1) the original proposals as produced during the five working weekends, 2) the clustered proposals carried out by the coordinating team, 3) the results of online members’ reviews of the proposals, 4) the submissions received from the public via the online form, and 5) the contact details of the experts met during the thematic cycles. The proposals made by the 6 groups were reviewed, refined, and advised by experts before being shared with all KBR members via the online collaboration platform Basecamp. At the end of this phase, the organizers grouped the recommendations. Then, the members had the opportunity to adopt (or not) each of the recommendations via an online (simple majority) vote that occurred during the first week of September 2022. When the final vote was opened to the 91 officially remaining assembly members during the first week of September 2022, 63 approved, the final recommendations online (69% participation rate). Each of the 56 proposed recommendations received a simple majority of the votes and were hence officially adopted by the KBR.

## Table 1A. The KBR timeline

| PHASE 1 |  |  |
| --- | --- | --- |
| 29.01.2022 | Kick-off event |  |
| 10.02.2022 | Optional online debate (I) |  |
| 14.02.2022 | Optional on-site visit | *Visit of Kass-Haff, Mersch* |
| 25.02.2022 | Optional on-site visit | *Visit of the project 2000m^2^, Kockelscheuer* |
| 25.02.2022 | Optional on-site visit | *“Ellergronn”, Esch-sur-Alzette* |
| **26-27.02.2022** | **WEEKEND 1. Agriculture and forestry** | At Neumünster Abbay, Luxembourg |
| 17.03.2022 | Optional online debate (II) |  |
| 25.03.2022 | Optional on-site visit | *Visit of “Energie- Atelier” of “Kanton Réiden”, Rédange-sur-Atert* |
| 25.03.2022 | Optional on-site visit | *Visit of la Soler’s “Wandpark Garnich”, Garnich* |
| 25.03.2022 | Optional on-site visit | *Visit of Schifflange, certified “Gold” within the ‘Pacte Climatique’ framework* |
| **26-27.03.2022** | **WEEKEND 1I. Renewable energy** | At “Foundry », Luxembourg |
| 21.04.2022 | Optional online debate (III) |  |
| 22.04.2022 | Optional on-site visit | *Visit of “Elmen”* |
| 22.04.2022 | Optional on-site visit | *Visit of the site “Neobuild”, Bettembourg* |
| **23-24.03.2022** | **WEEKEND 3. Sustainable construction** | At SNHBM, the site of the IFSB, Capellen |
| 05.05.2022 | Optional online debate (IV) |  |
| 06.05.2022 | Optional on-site visit | *Visit of recycling center “PreZero Lamesch”, Bettembourg* |
| 07.05.2022 | Optional on-site visit | *Visit of the “Superdreckskëscht”, Colmar-Ber* |
| **14-15.05.2022** | **WEEKEND 4. Waste Management** | At Ecological center SISPOLO, Parc Hosingen |
| 02.06.2022 | Optional online debate (V) |  |
| 03.06.2022 | Optional on-site visit | *Visit of Emile Weber’s company, Canach* |
| 10.06.2022 | Optional on-site visit | *Visit of “Losch Digital Lab”, Kockelscheuer* |
| 11-12.06.2022 | **WEEKEND 5. Mobility and transport** | At “Mama Shelter”, Luxembourg |
| PHASE II |  |  |
| 30.06.2022 | Kick off meeting | Online |
| July-Aug. 2022 | 4 working group meetings | Self-organized, in person or online |
| 03-08.09.2022 | Final vote |  |
| **15.09.2022** | **Final report delivery and report** | Parliament, Luxembourg |

Outcomes: recommendations and their impact

The KBR effectively engaged the members in policy development, meaning that they were empowered to generate their own recommendations. The process resulted in 56 recommendations that were each approved by a majority of the members and centralized in a final report, which was presented to the Government on the 15^th^ of September 2022, and debate in Parliament on the 3^rd^ of October 2022. In May 2023, along with the presentation of the advances on the draft of the new NECP, the government released (and updated) an official public document presenting how it was responding and considering each of the 56 recommendations (which themselves could encompass a series of different measures). To sum up, the KBR made 56 recommendations, which ended up in a total number of 142 proposals of concrete measures. From the official response released by the government, we learned that:

- 14 measures were not able to be immediately implemented at the national level, because they felt within the scope of European law, or they could not be carried out without international cooperation.
- 71 measures went beyond the scope of the NECP and will thus be addressed via government policy in the form of plans, strategies, regulations, and laws, for example in the area of waste, sustainable development, and environmental protection.
- 33 measures were already existing in a similar form. The government has decided to analyze these measures and draw up an assessment to ensure that the objectives proposed by KBR are respected. If this is not the case, the government said that they will make modifications to the final version of the NECP.
- 19 measures were reinforcing existing measures, which Luxembourg will transpose at the national level.
- 5 measures were genuinely new, and Luxembourg will transpose them at the national level. They are presented in Table 22.

The main KBR stimulus was thus to reinforce Luxembourg’s commitment to certain aspects of its climate policy. Out of the 197 measures included in the new NECP, 57 can be traced back to some of the KBR recommendations. Among these 57 KBR measures present in the NECP, 5 are real new measures, which would thus not have been implemented without the KBR. They concentrated on the need to do more regarding climate-friendly mobility, also passing by a more sustainable urban planning approach to cities’ development.

# **Appendix B. Samples: representativeness and attrition across survey waves**

## Table 1B – Socio-demographic characteristics of the maxi-public sample in the two waves

|  | Population | Sample | | | | Attrition | |
| --- | --- | --- | --- | --- | --- | --- | --- |
|  | (%) | Pre (%) | Δ (pre -population) | Post (%) | Δ (post -population) | Δ post-pre | *P value* |
| **Gender** |  |  |  |  |  |  | *.019* |
| Female | 50.0 | 50.0 | = | 46.0 | -4.0 | -4.0 |  |
| Male | 50.0 | 50.0 | = | 54.0 | +4.0 | +4.0 |  |
| **Age** |  |  |  |  |  |  | *.022* |
| 16–24 years old | 12.0 | 10.0 | -2.0 | 9.0 | -3.0 | -1.0 |  |
| 25–34 years old | 19.0 | 17.0 | -2.0 | 15.0 | -4.0 | -2.0 |  |
| 35–44 years old | 18.0 | 18.0 | = | 18.0 | = | = |  |
| 45–54 years old | 18.0 | 17.0 | -1.0 | 18.0 | = | +1.0 |  |
| 55–64 years old | 15.0 | 18.0 | +3.0 | 19.0 | +4.0 | +1.0 |  |
| 65 years old + | 18.0 | 20.0 | +2.0 | 21.0 | +3.0 | +1.0 |  |
| **Nationality** |  |  |  |  |  |  | *.634* |
| National (only Luxembourg citizenship) | 53.0 | 66.0 | +13.0 | 67.0 | +14.0 | +1.0 |  |
| Non-national (Luxembourg and/or other citizenship(s)) | 47.0 | 34.0 | -13.0 | 33.0 | -14.0 | -1.0 |  |
| **Region** |  |  |  |  |  |  |  |
| Luxembourg-City | 20.0 | 17.0 | -3.0 | 20.0 | = | +3.0 | *.150* |
| Center | 16.0 | 16.0 | = | 16.0 | = | = |  |
| South | 37.0 | 37.0 | = | 37.0 | = | = |  |
| North | 15.0 | 16.0 | +1.0 | 15.0 | = | -1.0 |  |
| East | 12.0 | 14.0 | +2.0 | 12.0 | = | -2.0 |  |
| **Occupation** |  |  |  |  |  |  | *.326* |
| In paid work (active) | 57.0 | 53.0 | -4.0 | 55.0 | -2.0 | +2.0 |  |
| Not in paid work (inactive) | 43.0 | 47.0 | +4.0 | 45.0 | +2.0 | -2.0 |  |

Note: The figures presented in the table are rounded numbers. They are based on the full raw sample and population census provided by the survey company along with the report of their fieldwork. Comparing pre- and post-wave samples with the population census allows to assess their representativeness. Overall, except for nationality – yet a common bias when surveying the Luxembourg population, the biases are limited, with only older respondents (and hence inactive, potentially retired individuals) being slightly overrepresented. These biases were compensated with socio-demographic weights. The last column indicates the difference in percentage points between the pre- and post-wave samples. Attrition was more pronounced among older and female respondents.

## Table 2B – Socio-demographic characteristics of the mini-public sample in the two waves

|  | Population | Sample | | | | Attrition | |
| --- | --- | --- | --- | --- | --- | --- | --- |
|  | (%) | Pre (%) | Δ (pre -population) | Post (%) | Δ (post -population) | Δ post-pre | *P value* |
| **Gender** |  |  |  |  |  |  | *.935* |
| Female | 50.0 | 50.0 | = | 50.0 | = | = |  |
| Male | 50.0 | 50.0 | = | 50.0 | = | = |  |
| **Age** |  |  |  |  |  |  | *.021* |
| 16–24 years old | 12.0 | 16.0 | +4.0 | 4.0 | -8.0 | -12.0 |  |
| 25–34 years old | 19.0 | 14.0 | -5.0 | 17.0 | -2.0 | +3.0 |  |
| 35–44 years old | 19.0 | 22.0 | +3.0 | 17.0 | -2.0 | -5.0 |  |
| 45–54 years old | 18.0 | 22.0 | +4.0 | 20.0 | +2.0 | -2.0 |  |
| 55–64 years old | 15.0 | 14.0 | -1.0 | 21.0 | +6.0 | +7.0 |  |
| 65 years old + | 18.0 | 12.0 | -6.0 | 21.0 | +3.0 | +9.0 |  |
| **Nationality** |  |  |  |  |  |  | *.516* |
| National (only Luxembourg citizenship) | 53.0 | 48.0 | -5.0 | 54.0 | +1.0 | +6.0 |  |
| Non-national (Luxembourg and/or other citizenship(s)) | 47.0 | 52.0 | +5.0 | 46.0 | -6.0 | -6.0 |  |
| **Occupation** |  |  |  |  |  |  | *.873* |
| In paid work (active) | 57.0 | 52.0 | -5.0 | 54.0 | -3.0 | +2.0 |  |
| Not in paid work (inactive) | 43.0 | 48.0 | +5.0 | 46.0 | +3.0 | -2.0 |  |

Note: The figures presented in the table are rounded. They are based on the distribution of the full raw sample of KBR participants and population census data. The samples of participants are not fully representative of the population, as not all KBR participants responded to our surveys. Moreover, the small number of observations implies that percentage points can change quickly. Attrition is therefore more pronounced. We can clearly see that younger respondents were more difficult to retain in the post-wave (the *p*-value of the *t*-test is statistically significant).

## Table 3B – Output of logisitic regression predicting probability of dropping out in both samples

|  | **All** |  | **Maxi-public only** | | **Mini public only** | |
| --- | --- | --- | --- | --- | --- | --- |
|  | Odds ratio | p value | Odds ratio | p value | Odds ratio | p value |
| Gender | .7517156 | 0.000 | .748326 | 0.000 | .9699331 | 0.956 |
| Age | .9256544 | 0.001 | .9345502 | 0.004 | .5975881 | 0.008 |
| Nationality | .9535073 | 0.540 | .9684146 | 0.684 | .7530434 | 0.600 |
| Occupation | 1.010496 | 0.894 | 1.039486 | 0.626 | .3675129 | 0.079 |
| Participant | 1.253577 | 0.364 |  |  |  |  |
| Constant | 1.103209 | 0.429 | 1.045797 | 0.722 | 10.39901 | 0.006 |
| Pseudo R2 | 0.0088 |  | 0.0080 |  | 0.1242 |  |

# **Appendix C. Questions’ wording, variables’ operationalization and descriptive statistics**

## Table 1C – Descriptive Statistics

|  | | | **Pre** | | | | | | | | | **Post** | | | | | | | | |  |  |
| --- | --- | --- | --- | --- | --- | --- | --- | --- | --- | --- | --- | --- | --- | --- | --- | --- | --- | --- | --- | --- | --- | --- |
| **Variables** | | **Question and answers** (DK and NA not displayed) | No exposure to KBR | | Exposure to KBR | | | Participants | | | No exposure to KBR | | | | Exposure to KBR | | | Participants | | |  |  |
|  | | **N** | N | % | N | % | N | | % | N | | | % | N | | % | N | | % |  |  |  |
| **Dependent variables** | **Support for more frequent DMPs** | How much do you agree with the following statement? Citizens' Assemblies like the KBR should be organized on other issues |  | | | | | | | | | | | | | | | | | |  |  |
|  |  |  |  |  |  |  |  |  |  |  |  |  |  |  |  |  |  |  |  |  |  | |
|  |  | Fully disagree = 1 | 4 | 1.4 | 26 | 1.8 | 0 | | 0 | 8 | | | 2.8 | 43 | | 3.1 | 1 | | 1.9 |  | |  |
|  |  | Disagree = 2 | 7 | 2.5 | 56 | 4 | 2 | | 2.7 | 9 | | | 3.2 | 61 | | 4.4 | 1 | | 1.9 |  | |  |
|  |  | Neutral = 3 | 69 | 24.4 | 259 | 18.2 | 7 | | 9.6 | 74 | | | 25.8 | 246 | | 17.5 | 7 | | 13.5 |  | |  |
|  |  | Agree = 4 | 138 | 48.7 | 758 | 53.3 | 28 | | 38.4 | 151 | | | 52.8 | 751 | | 53.5 | 16 | | 30.8 |  | |  |
|  |  | Fully agree = 5 | 65 | 23 | 323 | 22.7 | 36 | | 49.3 | 44 | | | 15.4 | 301 | | 21.5 | 27 | | 51.9 |  | |  |
|  |  | Mean | 3.89 |  | 3.91 |  | 4.34 | |  | 3.75 | | |  | 3.89 | |  | 4.29 | |  |  | |  |
|  |  | Total | 283 | 100 | 1422 | 100 | 73 | | 100 | 286 | | | 100 | 1402 | | 100 | 52 | | 100 |  | |  |
|  | **Participation in DMPs** | If retained to be a member of a Citizens' Assembly or public consultation on climate change or any other important issue in the future, how likely is it that you would participate? |  | | | | | | | | | | | | | | | | | |  | |
|  |  |  |  |  |  |  |  |  |  |  |  |  |  |  |  |  |  |  |  |  |  | |
|  |  | 0 very unlikely | 32 | 11.5 | 112 | 8.1 | 0 | | 0 | 55 | | | 19.2 | 132 | | 9.5 | 0 | | 0 |  | |  |
|  |  | 1 | 22 | 7.9 | 76 | 5.5 | 0 | | 0 | 20 | | | 7 | 87 | | 6.3 | 0 | | 0 |  | |  |
|  |  | 2 | 30 | 10.8 | 60 | 4.3 | 0 | | 0 | 26 | | | 9.1 | 78 | | 5.6 | 0 | | 0 |  | |  |
|  |  | 3 | 27 | 9.7 | 97 | 7.0 | 0 | | 0 | 30 | | | 10.5 | 108 | | 7.8 | 0 | | 0 |  | |  |
|  |  | 4 | 19 | 6.8 | 75 | 5.4 | 1 | | 1.408451 | 24 | | | 8.4 | 68 | | 4.9 | 0 | | 0 |  | |  |
|  |  | 5 | 41 | 14.7 | 136 | 9.8 | 4 | | 5.633803 | 38 | | | 13.3 | 145 | | 10.4 | 2 | | 3.8 |  | |  |
|  |  | 6 | 23 | 8.3 | 170 | 12.3 | 3 | | 4.225352 | 26 | | | 9.1 | 125 | | 9 | 6 | | 11.5 |  | |  |
|  |  | 7 | 38 | 13.7 | 201 | 14.5 | 14 | | 19.71831 | 20 | | | 7 | 227 | | 16.3 | 7 | | 13.5 |  | |  |
|  |  | 8 | 20 | 7.2 | 198 | 14.3 | 15 | | 21.126761 | 21 | | | 7.3 | 173 | | 12.5 | 8 | | 15.4 |  | |  |
|  |  | 9 | 5 | 1.8 | 101 | 7.3 | 13 | | 18.309859 | 8 | | | 2.8 | 99 | | 7.1 | 15 | | 28.8 |  | |  |
|  |  | 10 very likely | 21 | 7.6 | 159 | 11.5 | 21 | | 29.577465 | 18 | | | 6.3 | 147 | | 10.6 | 14 | | 26.9 |  | |  |
|  |  | Mean | 4.54 |  | 5.76 |  | 8.27 | |  | 4.07 | | |  | 5.51 | |  | 8.35 | |  |  | |  |
|  |  | Total | 278 | 100 | 1385 | 100 | 71 | | 100 | 290 | | | 100 | 1395 | | 100 | 60 | | 100 |  | |  |
|  | **Support for binding DMPs** | How would you evaluate these decision-making alternatives? *Decisions should be taken by a group of randomly selected citizens who gather for several days to deliberate on the issue* |  | | | | | | | | | | | | | | | | | |  | |
|  |  | 0 bad way of taking decisions | 0 | 0 | 0 | 0 | 15 | | 23.8 | 0 | | | 0 | 0 | | 0 | 13 | | 26 |  | |  |
|  |  | 1 | 58 | 19.1 | 300 | 20.8 | 13 | | 20.6 | 54 | | | 17.8 | 299 | | 20.7 | 4 | | 8 |  | |  |
|  |  | 2 | 27 | 8.9 | 192 | 13.3 | 5 | | 7.9 | 25 | | | 8.2 | 146 | | 10.1 | 7 | | 14 |  | |  |
|  |  | 3 | 25 | 8.2 | 183 | 12.7 | 5 | | 7.9 | 28 | | | 9.2 | 192 | | 13.3 | 9 | | 18 |  | |  |
|  |  | 4 | 34 | 11.2 | 186 | 12.9 | 2 | | 3.2 | 35 | | | 11.5 | 204 | | 14.1 | 4 | | 8 |  | |  |
|  |  | 5 | 29 | 9.5 | 149 | 10.3 | 11 | | 17.5 | 31 | | | 10.2 | 166 | | 11.5 | 7 | | 14 |  | |  |
|  |  | 6 | 63 | 20.7 | 167 | 11.6 | 3 | | 4.8 | 63 | | | 20.7 | 177 | | 12.2 | 3 | | 6 |  | |  |
|  |  | 7 | 36 | 11.8 | 124 | 8.6 | 5 | | 7.9 | 25 | | | 8.2 | 114 | | 7.9 | 2 | | 4 |  | |  |
|  |  | 8 | 17 | 5.6 | 80 | 5.5 | 3 | | 4.8 | 22 | | | 7.2 | 72 | | 5 | 1 | | 2 |  | |  |
|  |  | 9 | 3 | 1 | 34 | 2.4 | 0 | | 0 | 13 | | | 4.3 | 47 | | 3.3 | 0 | | 0 |  | |  |
|  |  | 10 good way of taking decisions | 12 | 3.9 | 30 | 2.1 | 1 | | 1.6 | 8 | | | 2.6 | 28 | | 2 | 0 | | 0 |  | |  |
|  |  | Mean | 4.57 |  | 4.05 |  | 2.98 | |  | 4.64 | | |  | 4.13 | |  | 2.72 | |  |  | |  |
|  |  | Total | 304 | 100 | 1445 | 100 | 63 | | 100 | 309 | | | 100 | 1449 | | 100 | 53 | | 100 |  | |  |
| **Mediators** | **Process’ evaluation** | How much do you agree with the following statement? The participants of citizens’ assemblies like the KBR are informed and skilled enough to contribute to policymaking on climate protection |  | | | | | | | | | | | | | | | | | |  | |
|  |  |  |  |  |  |  |  |  |  |  |  |  |  |  |  |  |  |  |  |  |  | |
|  |  | Fully disagree = 1 | 3 | 1.1 | 21 | 1.6 | 2 | | 3.2 | 5 | | | 1.9 | 24 | | 1.8 | 0 | | 0 |  | |  |
|  |  | Disagree = 2 | 12 | 4.6 | 51 | 3.8 | 2 | | 3.2 | 17 | | | 6.4 | 68 | | 5.2 | 4 | | 7.8 |  | |  |
|  |  | Neutral = 3 | 79 | 30.2 | 288 | 21.4 | 7 | | 11.1 | 83 | | | 31.4 | 283 | | 21.5 | 5 | | 9.8 |  | |  |
|  |  | Agree = 4 | 137 | 52.3 | 795 | 59.1 | 31 | | 49.2 | 138 | | | 52.3 | 813 | | 61.9 | 29 | | 56.9 |  | |  |
|  |  | Fully agree = 5 | 31 | 11.8 | 190 | 14.1 | 21 | | 33.3 | 21 | | | 8 | 126 | | 9.6 | 13 | | 25.5 |  | |  |
|  |  | Mean | 3.69 |  | 3.8 |  | 4.06 | |  | 3.58 | | |  | 3.72 | |  | 4 | |  |  | |  |
|  |  | Total | 262 | 100 | 1345 | 100 | 63 | | 100 | 264 | | | 100 | 1314 | | 100 | 55 | | 100 |  | |  |
|  | **Outcomes’ evaluation** | How much do you agree with the following statement? I am willing to accept the policy recommendations produced by a citizens’ assembly like the KBR |  | | | | | | | | | | | | | | | | | |  | |
|  |  |  |  |  |  |  |  |  |  |  |  |  |  |  |  |  |  |  |  |  |  | |
|  |  | Fully disagree = 1 | 11 | 4.1 | 50 | 3.8 | 0 | | 0 | 14 | | | 5.3 | 78 | | 5.8 | 0 | | 0 |  | |  |
|  |  | Disagree = 2 | 20 | 7.5 | 122 | 9.2 | 1 | | 1.6 | 34 | | | 13 | 181 | | 13.5 | 1 | | 2 |  | |  |
|  |  | Neutral = 3 | 117 | 43.8 | 507 | 38.2 | 7 | | 10.9 | 111 | | | 42.4 | 470 | | 35 | 7 | | 13.7 |  | |  |
|  |  | Agree = 4 | 107 | 40.1 | 575 | 43.3 | 40 | | 62.5 | 93 | | | 35.5 | 543 | | 40.4 | 30 | | 58.8 |  | |  |
|  |  | Fully agree = 5 | 12 | 4.5 | 73 | 5.5 | 16 | | 25 | 10 | | | 3.8 | 71 | | 5.3 | 13 | | 25.5 |  | |  |
|  |  | Mean | 3.33 |  | 3.38 |  | 4.11 | |  | 3.19 | | |  | 3.26 | |  | 4.08 | |  |  | |  |
|  |  | Total | 267 | 100 | 1327 | 100 | 64 | | 100 | 262 | | | 100 | 1343 | | 100 | 51 | | 100 |  | |  |
| **Controls** | **Age** | What is your age? |  |  |  |  |  | |  |  | | |  |  | |  |  | |  |  | |  |
|  |  | 16-24 years old | 46 | 15.1 | 115 | 8 | 11 | | 13.6 | ---- | | | ---- | ---- | | ---- | ---- | | ---- |  | |  |
|  |  | 25-34 years old | 60 | 19.7 | 208 | 14.4 | 11 | | 13.6 | ---- | | | ---- | ---- | | ---- | ---- | | ---- |  | |  |
|  |  | 35-44 years old | 62 | 20.4 | 242 | 16.7 | 16 | | 19.8 | ---- | | | ---- | ---- | | ---- | ---- | | ---- |  | |  |
|  |  | 45-54 years old | 46 | 15.1 | 259 | 17.9 | 17 | | 21 | ---- | | | ---- | ---- | | ---- | ---- | | ---- |  | |  |
|  |  | 55-64 years old | 54 | 17.8 | 273 | 18.9 | 12 | | 14.8 | ---- | | | ---- | ---- | | ---- | ---- | | ---- |  | |  |
|  |  | 65-74 years old | 26 | 8.6 | 244 | 16.9 | 13 | | 16 | ---- | | | ---- | ---- | | ---- | ---- | | ---- |  | |  |
|  |  | 75 years old or more | 10 | 3.3 | 104 | 7.2 | 1 | | 1.2 | ---- | | | ---- | ---- | | ---- | ---- | | ---- |  | |  |
|  |  | Total | 304 | 100 | 1445 | 100 | 81 | | 100 |  | | |  |  | |  |  | |  |  | |  |
|  | **Gender** | Are you male or female? |  |  |  |  |  | |  |  | | |  |  | |  |  | |  |  | |  |
|  |  | Male | 127 | 41.8 | 818 | 56.6 | 40 | | 47.6 | ---- | | | ---- | ---- | | ---- | ---- | | ---- |  | |  |
|  |  | Female | 177 | 58.2 | 627 | 43.4 | 44 | | 52.4 | ---- | | | ---- | ---- | | ---- | ---- | | ---- |  | |  |
|  |  | Total | 304 | 100 | 1445 | 100 | 84 | | 100 |  | | |  |  | |  |  | |  |  | |  |
|  | **Education** | What is the highest level of education you have obtained until now? |  |  |  |  |  | |  |  | | |  |  | |  |  | |  |  | |  |
|  |  | Lower secondary education or below | 38 | 13 | 138 | 9.7 | 6 | | 7.4 | ---- | | | ---- | ---- | | ---- | ---- | | ---- |  | |  |
|  |  | Short-cycle tertiary education | 176 | 60 | 704 | 49.2 | 25 | | 30.9 | ---- | | | ---- | ---- | | ---- | ---- | | ---- |  | |  |
|  |  | Bachelor or more | 79 | 27 | 588 | 41.1 | 50 | | 61.7 | ---- | | | ---- | ---- | | ---- | ---- | | ---- |  | |  |
|  |  | Total | 293 | 100 | 1430 | 100 | 81 | | 100 | ---- | | | ---- | ---- | | ---- | ---- | | ---- |  | |  |
|  | **Climate skepticism** | How much do you agree with the following statement? Claims that human activities are changing the climate are exaggerated |  | | | | | | | | | | | | | | | | | |  | |
|  |  |  |  |  |  |  |  |  |  |  |  |  |  |  |  |  |  |  |  |  |  | |
|  |  | Fully disagree = 1 | 48 | 16.7 | 315 | 22 | 32 | | 48.5 | 49 | | | 17 | 318 | | 22.3 | 26 | | 51 |  | |  |
|  |  | Disagree = 2 | 111 | 38.5 | 650 | 45.4 | 29 | | 43.9 | 113 | | | 39.1 | 658 | | 46.1 | 14 | | 27.5 |  | |  |
|  |  | Neutral = 3 | 62 | 21.5 | 222 | 15.5 | 3 | | 4.5 | 55 | | | 19 | 184 | | 12.9 | 7 | | 13.6 |  | |  |
|  |  | Agree = 4 | 51 | 17.7 | 164 | 11.4 | 2 | | 3 | 52 | | | 18 | 190 | | 13.3 | 3 | | 5.9 |  | |  |
|  |  | Fully agree = 5 | 16 | 5.6 | 82 | 5.7 | 0 | | 0 | 20 | | | 6.9 | 78 | | 5.5 | 1 | | 2 |  | |  |
|  |  | Mean | 2.57 |  | 2.34 |  | 1.62 | |  | 2.59 | | |  | 2.34 | |  | 1.8 | |  |  | |  |
|  |  | Total | 288 | 100 | 1433 | 100 | 66 | | 99.9 | 289 | | | 100 | 1428 | | 100.1 | 51 | | 100 |  | |  |

# **Appendix D – Comparing groups: no exposure versus indirect exposure**

In the following table we replicate the analyses of the main text, but we compare citizens who were not aware of the KBR during the whole process and citizens who became aware of the process.

**Replication of Figure 3**

The table below shows the descriptive statistics of all analyses variables. The overall sample size is 1749. There were 17.32% cases with missing data. Complete case analysis was used for the subsequent mediation analysis.

Table 1D – Descriptive statistics of continuous variables (DMP support)

| Variables | N | Mean | SD | Median | IQR |
| --- | --- | --- | --- | --- | --- |
|  | | | | | |
| Organization of other DMPs | 1446 | 0.710 | 0.225 | 0.750 | 0.250 |
|  | 1446 | 3.264 | 0.941 | 3.000 | 1.000 |
| Quality of Participants | 1446 | 3.707 | 0.781 | 4.000 | 1.000 |
| Exposure to KBR | 1446 | 0.844 | 0.363 | 1.000 | 0.000 |
| Age | 1446 | 3.991 | 1.744 | 4.000 | 2.000 |
| Climate Scepticism | 1446 | 2.483 | 1.229 | 2.000 | 1.000 |
| Political interest | 1446 | 2.062 | 0.806 | 2.000 | 1.000 |
|  | | | | | |

## Table 2D – Descriptive statistics of categorical variables (DMP support)

| Variables | Levels | N | % |
| --- | --- | --- | --- |
|  | | | |
| Gender | Men | 826 | 57.123 |
|  | Women | 620 | 42.877 |
|  |  | | |
| Education | Lower secondary education | 143 | 9.889 |
|  | Short-cycle tertiary education | 735 | 50.830 |
|  | Bachelor or more | 568 | 39.281 |
|  | | | |

#### Regression analysis

The table below shows the estimates from the key regression models for the mediation analysis.

- The outcome variable, Organization of other DMPs, was modelled with regression
- The mediator, Recommendations, was modelled with regression
- The mediator, Quality of Participants, was modelled with regression

The mediator models indicated that the exposure variable, Exposure to KBR, was

- not significantly associated with the mediator, Recommendations, b = 0.056, 95% CI = (-0.081, 0.192).
- significantly associated with the mediator, Quality of Participants, b = 0.120, 95% CI = (0.005, 0.235).

After accounting for the effect of the mediators , the outcome model indicated that the treatment variable, Exposure to KBR, was not significantly associated with Organization of other DMPs, b = 0.019, 95% CI = (-0.012, 0.050).

The outcome model also indicated that

- Recommendations is significantly associated with the outcome, Organization of other DMPs, b = 0.074, 95% CI = (0.061, 0.086).
- Quality of Participants is significantly associated with the outcome, Organization of other DMPs, b = 0.037, 95% CI = (0.022, 0.051).

The effect of Gender, Age, Education, Climate Scepticism and Political interest were adjusted for in the above analyses.

Table 3D – Results from key regression analyses (DMP support)

|  | Recommendations | | | Quality of Participants | | | Organization of other DMPs | | |
| --- | --- | --- | --- | --- | --- | --- | --- | --- | --- |
| Variables | b | 95% CI | p-value | b | 95% CI | p-value | b | 95% CI | p-value |
| (Intercept) | 4.013*** | (3.700, 4.326) | 0.000 | 3.866*** | (3.602, 4.129) | 0.000 | 0.327*** | (0.231, 0.422) | 0.000 |
| Age | -0.064*** | (-0.094, -0.035) | 0.000 | 0.025* | (0.001, 0.050) | 0.045 | -0.003 | (-0.010, 0.004) | 0.410 |
| Exposure to KBR | 0.056 | (-0.081, 0.192) | 0.423 | 0.120* | (0.005, 0.235) | 0.041 | 0.019 | (-0.012, 0.050) | 0.234 |
| Education: Bachelor or more (ref. Lower secondary) | -0.195* | (-0.367, -0.022) | 0.027 | -0.059 | (-0.204, 0.087) | 0.429 | 0.003 | (-0.036, 0.042) | 0.881 |
| Education: Short-cycle tert. Edu. (ref. Lower secondary) | -0.161 | (-0.326, 0.005) | 0.057 | -0.047 | (-0.186, 0.092) | 0.507 | 0.032 | (-0.006, 0.070) | 0.098 |
| Gender: Women ref. Men | 0.103* | (0.003, 0.202) | 0.043 | 0.075 | (-0.009, 0.158) | 0.079 | 0.028* | (0.005, 0.051) | 0.015 |
| Political interest | -0.025 | (-0.090, 0.039) | 0.441 | -0.055* | (-0.110, -0.001) | 0.046 | -0.015* | (-0.030, -0.001) | 0.041 |
| Climate Scepticism | -0.151*** | (-0.190, -0.111) | 0.000 | -0.093*** | (-0.126, -0.060) | 0.000 | 0.002 | (-0.007, 0.011) | 0.703 |
| Quality of Participants | - | - | - | - | - | - | 0.037*** | (0.022, 0.051) | 0.000 |
| Recommendations | - | - | - | - | - | - | 0.074*** | (0.061, 0.086) | 0.000 |

***p <=.001; ***p* <.01; **p*<.05

#### Mediation analysis

Mediation analysis was performed based on the counter-factual framework and the interventional effect (Vansteelandt and Daniel, 2017; Chan and Leung, 2020). The analysis was conducted in R using the intmed package (Chan and Leung, 2020) with 3000 simulations.

Results from the mediation analysis indicated that

- the indirect effect through Recommendations was non-significant, p = 0.413; 14.580% of the total effect was mediated through Recommendations.
- the indirect effect through Quality of Participants was statistically significant, p = 0.041; 14.180% of the total effect was mediated through Quality of Participants.
- the indirect effect through the dependence between mediators was non-significant, p = 0.983.
- the indirect effect through the interaction between mediators was non-significant, p = 0.983.
- the direct effect was non-significant, p = 0.226.
- the total effect was non-significant, p = 0.103.

## Table 4D – Mediation analysis results (DMP support)

|  | Estimates | 95% CI | p-value |
| --- | --- | --- | --- |
| Indirect effect mediated through Recommendations | 0.004 | (-0.006, 0.014) | 0.413 |
| Indirect effect mediated through Quality of Participants | 0.004* | (0.000, 0.010) | 0.041 |
| Indirect effect mediated through the dependence between mediators | 0.000 | (-0.006, 0.006) | 0.983 |
| Indirect effect mediated through the interaction between mediators | 0.000 | (0.000, 0.000) | 0.983 |
| Direct effect | 0.019 | (-0.012, 0.050) | 0.226 |
| Total effect | 0.027 | (-0.006, 0.061) | 0.103 |
| Proportion of effect mediated through Recommendations | 0.146 |  |  |
| Proportion of effect mediated through Quality of Participants | 0.142 |  |  |

***p <=.001; ***p* <.01; **p*<.05

**Replication of Figure 4**

#### Descriptive statistics

The table below shows the descriptive statistics of all analyses variables. The overall sample size is 1749. There were 19.44% cases with missing data. Complete case analysis was used for the subsequent mediation analysis.

Table 5D – Descriptive statistics of continuous variables (DMP participation)

| Variables | N | Mean | SD | Median | IQR |
| --- | --- | --- | --- | --- | --- |
|  | | | | | |
| Willingness to Participate | 1409 | 0.541 | 0.309 | 0.600 | 0.500 |
| Recommendations | 1409 | 3.265 | 0.937 | 3.000 | 1.000 |
| Quality of Participants | 1409 | 3.716 | 0.772 | 4.000 | 1.000 |
| Exposure to KBR | 1409 | 0.847 | 0.360 | 1.000 | 0.000 |
| Age | 1409 | 3.991 | 1.749 | 4.000 | 2.000 |
| Climate Scepticism | 1409 | 2.477 | 1.224 | 2.000 | 1.000 |
| Political interest | 1409 | 2.061 | 0.805 | 2.000 | 1.000 |
|  | | | | | |

## Table 6D – Descriptive statistics of categorical variables (DMP participation)

| Variables | Levels | N | % |
| --- | --- | --- | --- |
|  | | | |
| Gender | Men | 802 | 56.920 |
|  | Women | 607 | 43.080 |
|  |  | | |
| Education | Lower secondary education | 137 | 9.723 |
|  | Short-cycle tertiary education | 712 | 50.532 |
|  | Bachelor or more | 560 | 39.744 |
|  | | | |

#### Regression analysis

The table below shows the estimates from the key regression models for the mediation analysis.

- The outcome variable, Willingness to Participate, was modelled with regression
- The mediator, Recommendations, was modelled with regression
- The mediator, Quality of Participants, was modelled with regression

The mediator models indicated that the exposure variable, Exposure to KBR, was

- not significantly associated with the mediator, Recommendations, b = 0.057, 95% CI = (-0.082, 0.195).
- significantly associated with the mediator, Quality of Participants, b = 0.142, 95% CI = (0.026, 0.257).

After accounting for the effect of the mediators , the outcome model indicated that the treatment variable, Exposure to KBR, was not significantly associated with Willingness to Participate, b = 0.043, 95% CI = (0.000, 0.086).

The outcome model also indicated that

- Recommendations is significantly associated with the outcome, Willingness to Participate, b = 0.047, 95% CI = (0.030, 0.064).
- Quality of Participants is not significantly associated with the outcome, Willingness to Participate, b = 0.020, 95% CI = (0.000, 0.040).

The effect of Gender, Age, Education, Climate Scepticism and Political interest were adjusted for in the above analyses.

Table 7D – Results from key regression analyses (DMP participation)

|  | Recommendations | | | Quality of Participants | | | Willingness to participate | | |
| --- | --- | --- | --- | --- | --- | --- | --- | --- | --- |
| Variables | b | 95% CI | p-value | b | 95% CI | p-value | b | 95% CI | p-value |
| (Intercept) | 3.976*** | (3.658, 4.294) | 0.000 | 3.868*** | (3.602, 4.134) | 0.000 | 0.533*** | (0.401, 0.665) | 0.000 |
| Age | -0.061*** | (-0.091, -0.032) | 0.000 | 0.019 | (-0.006, 0.043) | 0.145 | -0.025*** | (-0.035, -0.016) | 0.000 |
| Exposure to KBR | 0.057 | (-0.082, 0.195) | 0.422 | 0.142* | (0.026, 0.257) | 0.017 | 0.043* | (0.000, 0.086) | 0.050 |
| Education: Bachelor or more (ref. Lower secondary) | -0.178* | (-0.353, -0.003) | 0.047 | -0.043 | (-0.189, 0.104) | 0.565 | 0.146*** | (0.091, 0.200) | 0.000 |
| Education: Short-cycle tert. Edu. (ref. Lower secondary) | -0.141 | (-0.309, 0.027) | 0.100 | -0.026 | (-0.166, 0.115) | 0.718 | 0.055* | (0.003, 0.107) | 0.038 |
| Gender: Women ref. Men | 0.108* | (0.007, 0.208) | 0.035 | 0.057 | (-0.027, 0.141) | 0.180 | -0.025 | (-0.056, 0.007) | 0.122 |
| Political interest | -0.019 | (-0.085, 0.046) | 0.562 | -0.060* | (-0.115, -0.005) | 0.031 | -0.107*** | (-0.127, -0.086) | 0.000 |
| Climate Scepticism | -0.153*** | (-0.193, -0.113) | 0.000 | -0.087*** | (-0.121, -0.054) | 0.000 | -0.005 | (-0.017, 0.008) | 0.469 |
| Quality of Participants | - | - | - | - | - | - | 0.020 | (0.000, 0.040) | 0.051 |
| Recommendations | - | - | - | - | - | - | 0.047*** | (0.030, 0.064) | 0.000 |

***p <=.001;***p* <.01;**p*<.05.

#### Mediation analysis

Mediation analysis was performed based on the counter-factual framework and the interventional effect (Vansteelandt and Daniel, 2017; Chan and Leung, 2020). The analysis was conducted in R using the *intmed* package (Chan and Leung, 2020) with 3000 simulations.

Results from the mediation analysis indicated that

- the indirect effect through Recommendations was non-significant, p = 0.413; 5.268% of the total effect was mediated through Recommendations.
- the indirect effect through Quality of Participants was non-significant, p = 0.062; 5.380% of the total effect was mediated through Quality of Participants.
- the indirect effect through the dependence between mediators was non-significant, p = 0.995.
- the indirect effect through the interaction between mediators was non-significant, p = 0.997.
- the direct effect was non-significant, p = 0.053.
- the total effect was statistically significant, p = 0.031.

## Table 8D – Mediation analysis results (DMP participation)

|  | Estimates | 95% CI | p-value |
| --- | --- | --- | --- |
| Indirect effect mediated through Recommendations | 0.003 | (-0.004, 0.010) | 0.413 |
| Indirect effect mediated through Quality of Participants | 0.003 | (0.000, 0.007) | 0.062 |
| Indirect effect mediated through the dependence between mediators | 0.000 | (-0.004, 0.004) | 0.995 |
| Indirect effect mediated through the interaction between mediators | 0.000 | (0.000, 0.000) | 0.997 |
| Direct effect | 0.042* | (-0.001, 0.085) | 0.05 |
| Total effect | 0.048* | (0.004, 0.090) | 0.031 |
| Proportion of effect mediated through Recommendations | 0.053 |  |  |
| Proportion of effect mediated through Quality of Participants | 0.054 |  |  |

***p <=.001; ***p* <.01; **p*<.05

**Replication of Figure 5**

#### Descriptive statistics

The table below shows the descriptive statistics of all analyses variables. The overall sample size is 1749. There were 16.58% cases with missing data. Complete case analysis was used for the subsequent mediation analysis.

Table 9D – Descriptive statistics of continuous variables (binding DMPs)

| Variables | N | Mean | SD | Median | IQR |
| --- | --- | --- | --- | --- | --- |
|  | | | | | |
| Binding DMPs | 1459 | 0.388 | 0.228 | 0.364 | 0.364 |
| Recommendations | 1459 | 3.261 | 0.941 | 3.000 | 1.000 |
| Quality of Participants | 1459 | 3.705 | 0.780 | 4.000 | 1.000 |
| Exposure to KBR | 1459 | 0.846 | 0.361 | 1.000 | 0.000 |
| Age | 1459 | 3.990 | 1.749 | 4.000 | 2.000 |
| Climate Scepticism | 1459 | 2.485 | 1.228 | 2.000 | 1.000 |
| Political interest | 1459 | 2.064 | 0.805 | 2.000 | 1.000 |
|  | | | | | |

## Table 10D – Descriptive statistics of categorical variables (binding DMPs)

| Variables | Levels | N | % |
| --- | --- | --- | --- |
|  | | | |
| Gender | Men | 832 | 57.025 |
|  | Women | 627 | 42.975 |
|  |  | | |
| Education | Lower secondary education | 144 | 9.870 |
|  | Short-cycle tertiary education | 739 | 50.651 |
|  | Bachelor or more | 576 | 39.479 |
|  | | | |

#### Regression analysis

The table below shows the estimates from the key regression models for the mediation analysis.

- The outcome variable, Binding DMPs, was modelled with regression
- The mediator, Recommendations, was modelled with regression
- The mediator, Quality of Participants, was modelled with regression

The mediator models indicated that the exposure variable, Exposure to KBR, was

- not significantly associated with the mediator, Recommendations, b = 0.053, 95% CI = (-0.083, 0.190).
- significantly associated with the mediator, Quality of Participants, b = 0.117, 95% CI = (0.002, 0.231).

After accounting for the effect of the mediators , the outcome model indicated that the treatment variable, Exposure to KBR, was not significantly associated with Binding DMPs, b = -0.028, 95% CI = (-0.060, 0.004).

The outcome model also indicated that

- Recommendations is significantly associated with the outcome, Binding DMPs, b = 0.059, 95% CI = (0.047, 0.072).
- Quality of Participants is not significantly associated with the outcome, Binding DMPs, b = -0.003, 95% CI = (-0.018, 0.012).

The effect of Gender, Age, Education, Climate Scepticism and Political interest were adjusted for in the above analyses.

Table 11D – Results from key regression analyses (binding DMPs)

|  | Recommendations | | | Quality of Participants | | | Binding DMPs | | |
| --- | --- | --- | --- | --- | --- | --- | --- | --- | --- |
| Variables | b | 95% CI | p-value | b | 95% CI | p-value | b | 95% CI | p-value |
| (Intercept) | 4.003*** | (3.691, 4.316) | 0.000 | 3.867*** | (3.605, 4.130) | 0.000 | 0.124* | (0.026, 0.223) | 0.013 |
| Age | -0.063*** | (-0.092, -0.034) | 0.000 | 0.024 | (-0.001, 0.049) | 0.055 | -0.013*** | (-0.020, -0.006) | 0.000 |
| Exposure to KBR | 0.053 | (-0.083, 0.190) | 0.444 | 0.117* | (0.002, 0.231) | 0.046 | -0.028 | (-0.060, 0.004) | 0.091 |
| Education: Bachelor or more (ref. Lower secondary) | -0.195* | (-0.367, -0.023) | 0.026 | -0.053 | (-0.198, 0.091) | 0.470 | 0.016 | (-0.024, 0.057) | 0.428 |
| Education: Short-cycle tert. Edu. (ref. Lower secondary) | -0.158 | (-0.322, 0.007) | 0.061 | -0.045 | (-0.183, 0.094) | 0.529 | 0.017 | (-0.022, 0.055) | 0.402 |
| Gender: Women ref. Men | 0.108* | (0.009, 0.207) | 0.032 | 0.072 | (-0.011, 0.155) | 0.090 | 0.015 | (-0.008, 0.039) | 0.197 |
| Political interest | -0.025 | (-0.090, 0.039) | 0.443 | -0.058* | (-0.112, -0.003) | 0.037 | 0.036*** | (0.021, 0.052) | 0.000 |
| Climate Scepticism | -0.150*** | (-0.190, -0.111) | 0.000 | -0.091*** | (-0.124, -0.057) | 0.000 | 0.024*** | (0.015, 0.034) | 0.000 |
| Quality of Participants | - | - | - | - | - | - | -0.003 | (-0.018, 0.012) | 0.673 |
| Recommendations | - | - | - | - | - | - | 0.059*** | (0.047, 0.072) | 0.000 |

***p <=.001;***p* <.01;**p* <.05.

Mediation analysis

Mediation analysis was performed based on the counter-factual framework and the interventional effect (Vansteelandt and Daniel, 2017; Chan and Leung, 2020). The analysis was conducted in R using the intmed package (Chan and Leung, 2020) with 3000 simulations.
Results from the mediation analysis indicated that

- the indirect effect through Recommendations was non-significant, p = 0.429; -9.114% of the total effect was mediated through Recommendations.
- the indirect effect through Quality of Participants was non-significant, p = 0.663; 0.981% of the total effect was mediated through Quality of Participants.
- the indirect effect through the dependence between mediators was non-significant, p = 0.999.
- the indirect effect through the interaction between mediators was non-significant, p = 0.999.
- the direct effect was non-significant, p = 0.087.
- the total effect was non-significant, p = 0.143.

## Table 12D – Mediation analysis results (binding DMPs)

|  | Estimates | 95% CI | p-value |
| --- | --- | --- | --- |
| Indirect effect mediated through Recommendations | 0.003 | (-0.005, 0.011) | 0.429 |
| Indirect effect mediated through Quality of Participants | 0.000 | (-0.003, 0.002) | 0.663 |
| Indirect effect mediated through the dependence between mediators | 0.000 | (-0.001, 0.002) | 0.999 |
| Indirect effect mediated through the interaction between mediators | 0.000 | (0.000, 0.000) | 0.999 |
| Direct effect | -0.028 | (-0.059, 0.005) | 0.087 |
| Total effect | -0.025 | (-0.057, 0.009) | 0.143 |
| Proportion of effect mediated through Recommendations | -0.091 |  |  |
| Proportion of effect mediated through Quality of Participants | 0.010 |  |  |

***p <=.001; ***p* <.01; **p*<.05.

# **Appendix E – Comparing groups: indirect exposure versus direct exposure**

In the following table we replicate the analyses of the main text, but we compare citizens who were exposed to KBR, but did not participate in it (“Exposure to KBR”) and KBR participants (“Participation in KBR”).

**Replication of Figure 3**

#### Descriptive statistics

The table below shows the descriptive statistics of all analyses variables. The overall sample size is 1537. There were 18.02% cases with missing data. Complete case analysis wass used for the subsequent mediation analysis.

Table 1E – Descriptive statistics of continuous variables (DMP support)

| Variables | N | Mean | SD | Median | IQR |
| --- | --- | --- | --- | --- | --- |
|  | | | | | |
| Organization of other DMPs | 1260 | 0.719 | 0.227 | 0.750 | 0.000 |
| Recommendations | 1260 | 3.301 | 0.957 | 3.000 | 1.000 |
| Quality of Participants | 1260 | 3.745 | 0.773 | 4.000 | 1.000 |
| Exposure to KBR | 1260 | 0.031 | 0.173 | 0.000 | 0.000 |
| Age | 1260 | 4.085 | 1.729 | 4.000 | 3.000 |
| Climate Scepticism | 1260 | 2.405 | 1.235 | 2.000 | 2.000 |
| Political interest | 1260 | 1.960 | 0.755 | 2.000 | 1.000 |
|  | | | | | |

## Table 2E – Descriptive statistics of categorical variables (DMP support)

| Variables | Levels | N | % |
| --- | --- | --- | --- |
|  | | | |
| Gender | Men | 745 | 59.127 |
|  | Women | 515 | 40.873 |
|  |  | | |
| Education | Lower secondary education | 119 | 9.444 |
|  | Short-cycle tertiary education | 604 | 47.937 |
|  | Bachelor or more | 537 | 42.619 |
|  | | | |

#### Regression analysis

The table below shows the estimates from the key regression models for the mediation analysis.

- The outcome variable, Organization of other DMPs, was modelled with regression
- The mediator, Recommendations, was modelled with regression
- The mediator, Quality of Participants, was modelled with regression

The mediator models indicated that the exposure variable, Exposure to KBR, was

- significantly associated with the mediator, Recommendations, b = 0.753, 95% CI = (0.457, 1.048).
- not significantly associated with the mediator, Quality of the Participants, b = 0.209, 95% CI = (-0.038, 0.456).

After accounting for the effect of the mediators , the outcome model indicated that the treatment variable, Exposure to KBR, was significantly associated with Organization of other DMPs, b = 0.069, 95% CI = (0.001, 0.137).

The outcome model also indicated that

- Recommendations is significantly associated with the outcome, Organization of other DMPs, b= 0.080, 95% CI = (0.066, 0.093).
- Quality of Participants is significantly associated with the outcome, Organization of other DMPs, b = 0.029, 95% CI = (0.013, 0.044).

The effect of Gender, Age, Education, Climate Scepticism and Political interest were adjusted for in the above analyses.

## Table 3E. Results from key regression analyses (DMP support)

|  | Recommendations | | | Quality of Participants | | | Organization of other DMPs | | |
| --- | --- | --- | --- | --- | --- | --- | --- | --- | --- |
| Variables | b | 95% CI | p-value | b | 95% CI | p-value | b | 95% CI | p-value |
| (Intercept) | 4.027*** | (3.725, 4.329) | 0.000 | 3.950*** | (3.698, 4.202) | 0.000 | 0.355*** | (0.257, 0.452) | 0.000 |
| Age | -0.059*** | (-0.090, -0.027) | 0.000 | 0.030* | (0.004, 0.056) | 0.026 | -0.004 | (-0.011, 0.003) | 0.301 |
| Exposure to KBR | 0.753*** | (0.457, 1.048) | 0.000 | 0.209 | (-0.038, 0.456) | 0.098 | 0.069* | (0.001, 0.137) | 0.047 |
| Education: Bachelor or more (ref. Lower secondary) | -0.189* | (-0.376, -0.002) | 0.048 | -0.078 | (-0.235, 0.078) | 0.325 | 0.001 | (-0.042, 0.043) | 0.972 |
| Education: Short-cycle tert. Edu. (ref. Lower secondary) | -0.131 | (-0.312, 0.050) | 0.156 | -0.037 | (-0.189, 0.114) | 0.630 | 0.028 | (-0.014, 0.069) | 0.189 |
| Gender: Women ref. Men | 0.140** | (0.034, 0.247) | 0.010 | 0.046 | (-0.043, 0.135) | 0.308 | 0.035** | (0.011, 0.059) | 0.005 |
| Political interest | -0.019 | (-0.090, 0.052) | 0.599 | -0.043 | (-0.103, 0.016) | 0.150 | -0.014 | (-0.031, 0.002) | 0.082 |
| Climate Scepticism | -0.161*** | (-0.203, -0.118) | 0.000 | -0.090*** | (-0.126, -0.054) | 0.000 | 0.003 | (-0.007, 0.013) | 0.498 |
| Quality of Participants | - | - | - | - | - | - | 0.029*** | (0.013, 0.044) | 0.000 |
| Recommendations | - | - | - | - | - | - | 0.080*** | (0.066, 0.093) | 0.000 |

***p <=.001; ***p* <.01; **p*<.05.

#### Mediation analysis

Mediation analysis was performed based on the counter-factual framework and the interventional effect (Vansteelandt and Daniel, 2017; Chan and Leung, 2020). The analysis was conducted in R using the intmed package (Chan and Leung, 2020) with 3000 simulations.

Results from the mediation analysis indicated that

- the indirect effect through Recommendations was non-significant, p=0.000; 44.107% of the total effect was mediated through Recommendations.
- the indirect effect through Quality of Participants was statistically significant, p = 0.097; 4.153% of the total effect was mediated through Quality of Participants.
- the indirect effect through the dependence between mediators was non-significant, p = 0.983.
- the indirect effect through the interaction between mediators was non-significant, p = 0.983.
- the direct effect was statistically significant, p = 0.047.
- the total effect was statistically significant, p = 0.000.

## Table 4E – Mediation analysis results (DMP support)

|  | Estimates | 95% CI | p-value |
| --- | --- | --- | --- |
| Indirect effect mediated through Recommendations | 0.060*** | (0.035, 0.087) | 0.000 |
| Indirect effect mediated through Quality of Participants | 0.006 | (-0.001, 0.015) | 0.097 |
| Indirect effect mediated through the dependence between mediators | 0.000 | (-0.011, 0.011) | 0.989 |
| Indirect effect mediated through the interaction between mediators | 0.000 | (0.000, 0.000) | 0.977 |
| Direct effect | 0.070* | (0.001, 0.136) | 0.047 |
| Total effect | 0.136*** | (0.061, 0.208) | 0.000 |
| Proportion of effect mediated through Recommendations | 0.441 |  |  |
| Proportion of effect mediated through Quality of Participants | 0.042 |  |  |

****p* < .001;***p* < .05****p* < .05.

**Replication of Figure 4**

#### Descriptive statistics

The table below shows the descriptive statistics of all analyses variables. The overall sample size is 1537. There were 19.84% cases with missing data. Complete case analysis was used for the subsequent mediation analysis.

Table 5E – Descriptive statistics of continuous variables (DMP participation)

| Variables | N | Mean | SD | Median | IQR |
| --- | --- | --- | --- | --- | --- |
|  | | | | | |
| Willingness to participate | 1232 | 0.568 | 0.307 | 0.600 | 0.500 |
| Recommendations | 1232 | 3.302 | 0.953 | 3.000 | 1.000 |
| Quality of Participants | 1232 | 3.756 | 0.764 | 4.000 | 1.000 |
| Exposure to KBR | 1232 | 0.032 | 0.175 | 0.000 | 0.000 |
| Age | 1232 | 4.090 | 1.736 | 4.000 | 3.000 |
| Climate Scepticism | 1232 | 2.399 | 1.228 | 2.000 | 2.000 |
| Political interest | 1232 | 1.961 | 0.754 | 2.000 | 1.000 |
|  | | | | | |

## Table 6E – Descriptive statistics of categorical variables (DMP participation)

| Variables | Levels | N | % |
| --- | --- | --- | --- |
|  | | | |
| Gender | Men | 727 | 59.010 |
|  | Women | 505 | 40.990 |
|  |  | | |
| Education | Lower secondary education | 115 | 9.334 |
|  | Short-cycle tertiary education | 588 | 47.727 |
|  | Bachelor or more | 529 | 42.938 |
|  | | | |

#### Regression analysis

The table below shows the estimates from the key regression models for the mediation analysis.

- The outcome variable, Willingness to participate, was modelled with regression
- The mediator, Recommendations, was modelled with regression
- The mediator, Quality of Participants, was modelled with regression

The mediator models indicated that the exposure variable, Exposure to KBR, was

- significantly associated with the mediator, Recommendations, b = 0.750, 95% CI = (0.456, 1.044).
- not significantly associated with the mediator, Quality of Participants, b = 0.198, 95% CI = (-0.046, 0.443).

After accounting for the effect of the mediators , the outcome model indicated that the treatment variable, Exposure to KBR, was significantly associated with Willingness to participate, b = 0.174, 95% CI = (0.084, 0.265).

The outcome model also indicated that

- Recommendations is significantly associated with the outcome, Willingness to participate, b = 0.050, 95% CI = (0.032, 0.068).
- Quality of Participants is not significantly associated with the outcome, Willingness to participate, b = 0.019, 95% CI = (-0.003, 0.040).

The effect of Gender, Age, Education, Climate Scepticism and Political interest were adjusted for in the above analyses.

## Table. 7E – Results from key regression analyses (DMP participation)

|  | Recommendations | | | Quality of Participants | | | W3_QC7_1 | | |
| --- | --- | --- | --- | --- | --- | --- | --- | --- | --- |
| Variables | b | 95% CI | p-value | b | 95% CI | p-value | b | 95% CI | p-value |
| (Intercept) | 4.001*** | (3.696, 4.306) | 0.000 | 4.000*** | (3.746, 4.253) | 0.000 | 0.587*** | (0.455, 0.719) | 0.000 |
| Age | -0.055*** | (-0.087, -0.023) | 0.001 | 0.022 | (-0.005, 0.048) | 0.107 | -0.030*** | (-0.040, -0.020) | 0.000 |
| Exposure to KBR | 0.750*** | (0.456, 1.044) | 0.000 | 0.198 | (-0.046, 0.443) | 0.112 | 0.174*** | (0.084, 0.265) | 0.000 |
| Education: Bachelor or more (ref. Lower secondary) | -0.174 | (-0.363, 0.014) | 0.070 | -0.068 | (-0.225, 0.089) | 0.395 | 0.150*** | (0.092, 0.207) | 0.000 |
| Education: Short-cycle tert. Edu. (ref. Lower secondary) | -0.113 | (-0.296, 0.070) | 0.227 | -0.016 | (-0.168, 0.137) | 0.841 | 0.055 | (-0.001, 0.111) | 0.054 |
| Gender: Women ref. Men | 0.145** | (0.038, 0.252) | 0.008 | 0.028 | (-0.061, 0.117) | 0.542 | -0.016 | (-0.049, 0.017) | 0.335 |
| Political interest | -0.016 | (-0.088, 0.055) | 0.656 | -0.053 | (-0.113, 0.006) | 0.080 | -0.107*** | (-0.129, -0.085) | 0.000 |
| Climate Scepticism | -0.165*** | (-0.208, -0.122) | 0.000 | -0.087*** | (-0.123, -0.051) | 0.000 | -0.004 | (-0.017, 0.010) | 0.575 |
| Quality of Participants | - | - | - | - | - | - | 0.019 | (-0.003, 0.040) | 0.092 |
| Recommendations | - | - | - | - | - | - | 0.050*** | (0.032, 0.068) | 0.000 |

****p* < .001;***p* < .05****p* < .05.

#### Mediation analysis

Mediation analysis was performed based on the counter-factual framework and the interventional effect (Vansteelandt and Daniel 2017; Chan and Leung 2020). The analysis was conducted in R using the intmed package (Chan and Leung, 2020) with 3000 simulations.

Results from the mediation analysis indicated that

- the indirect effect through Recommendations was statistically significant, p = 0.000; 17.451% of the total effect was mediated through Recommendations.
- the indirect effect through Quality of Participants was non-significant, p = 0.183; 1.431% of the total effect was mediated through Quality of Participants.
- the indirect effect through the dependence between mediators was non-significant, p = 0.977.
- the indirect effect through the interaction between mediators was non-significant, p = 0.958.
- the direct effect was statistically significant, p = 0.001.
- the total effect was statistically significant, p = 0.000.

## Table 8E – Mediation analysis results (DMP participation)

|  | Estimates | 95% CI | p-value |
| --- | --- | --- | --- |
| Indirect effect mediated through Recommendations | 0.037*** | (0.019, 0.059) | 0.000 |
| Indirect effect mediated through Quality of Participants | 0.004 | (-0.001, 0.012) | 0.183 |
| Indirect effect mediated through the dependence between mediators | 0.000 | (-0.008, 0.008) | 0.977 |
| Indirect effect mediated through the interaction between mediators | 0.000 | (0.000, 0.000) | 0.958 |
| Direct effect | 0.173*** | (0.083, 0.257) | 0.001 |
| Total effect | 0.214*** | (0.123, 0.297) | 0.000 |
| Proportion of effect mediated through Recommendations | 0.175 |  |  |
| Proportion of effect mediated through Quality of Participants | 0.014 |  |  |

***p <=.001; ***p* <.01; **p*<.05

**Replication of Figure 5 (binding DMPs)**

#### Descriptive statistics

The table below shows the descriptive statistics of all analyses variables. The overall sample size is 1537. There were 17.24% cases with missing data. Complete case analysis was used for the subsequent mediation analysis.

Table 9E – Descriptive statistics of continuous variables (binding DMPs)

| Variables | N | Mean | SD | Median | IQR |
| --- | --- | --- | --- | --- | --- |
|  | | | | | |
| W3_QF1_5a | 1272 | 0.376 | 0.226 | 0.364 | 0.364 |
| Recommendations | 1272 | 3.296 | 0.958 | 3.000 | 1.000 |
| Quality of Participants | 1272 | 3.743 | 0.773 | 4.000 | 1.000 |
| Exposure to KBR | 1272 | 0.030 | 0.170 | 0.000 | 0.000 |
| Age | 1272 | 4.084 | 1.734 | 4.000 | 3.000 |
| Climate Scepticism | 1272 | 2.407 | 1.235 | 2.000 | 2.000 |
| Political interest | 1272 | 1.964 | 0.755 | 2.000 | 1.000 |
|  | | | | | |

## Table 10E – Descriptive statistics of categorical variables (binding DMPs)

| Variables | Levels | N | % |
| --- | --- | --- | --- |
|  | | | |
| Gender | Men | 751 | 59.041 |
|  | Women | 521 | 40.959 |
|  |  | | |
| Education | Lower secondary education | 120 | 9.434 |
|  | Short-cycle tertiary education | 608 | 47.799 |
|  | Bachelor or more | 544 | 42.767 |
|  | | | |

#### Regression analysis

The table below shows the estimates from the key regression models for the mediation analysis.

- The outcome variable, Binding DMPs, was modelled with regression
- The mediator, Recommendations, was modelled with regression
- The mediator, Quality of Participants, was modelled with regression

The mediator models indicated that the exposure variable, Exposure to KBR, was

- significantly associated with the mediator, Recommendations, b = 0.762, 95% CI = (0.463, 1.062).
- not significantly associated with the mediator, Quality of Participants, b = 0.237, 95% CI = (-0.014, 0.487).

After accounting for the effect of the mediators , the outcome model indicated that the treatment variable, Exposure to KBR, was significantly associated with Binding DMPs, b = -0.118, 95% CI = (-0.188, -0.047).

The outcome model also indicated that

- Recommendations is significantly associated with the outcome, Binding DMPs, b = 0.062, 95% CI = (0.049, 0.076).
- Quality of Participants is not significantly associated with the outcome, Binding DMPs, b = -0.006, 95% CI = (-0.022, 0.010).

The effect of Gender, Age, Education, Climate Scepticism and Political interest were adjusted for in the above analyses.

## Table 11E – Results from key regression analyses (binding DMPs)

|  | Recommendations | | | Quality of Participants | | | Binding DMPs | | |
| --- | --- | --- | --- | --- | --- | --- | --- | --- | --- |
| Variables | b | 95% CI | p-value | b | 95% CI | p-value | b | 95% CI | p-value |
| (Intercept) | 4.013*** | (3.712, 4.314) | 0.000 | 3.943*** | (3.692, 4.195) | 0.000 | 0.094 | (-0.005, 0.193) | 0.062 |
| Age | -0.058*** | (-0.089, -0.026) | 0.000 | 0.029* | (0.002, 0.055) | 0.032 | -0.013*** | (-0.020, -0.005) | 0.001 |
| Exposure to KBR | 0.762*** | (0.463, 1.062) | 0.000 | 0.237 | (-0.014, 0.487) | 0.064 | -0.118** | (-0.188, -0.047) | 0.001 |
| Education: Bachelor or more (ref. Lower secondary) | -0.189* | (-0.374, -0.003) | 0.047 | -0.070 | (-0.226, 0.085) | 0.375 | 0.013 | (-0.031, 0.056) | 0.568 |
| Education: Short-cycle tert. Edu. (ref. Lower secondary) | -0.128 | (-0.308, 0.053) | 0.165 | -0.034 | (-0.184, 0.117) | 0.660 | 0.002 | (-0.040, 0.044) | 0.926 |
| Gender: Women ref. Men | 0.147** | (0.041, 0.252) | 0.007 | 0.044 | (-0.045, 0.132) | 0.331 | 0.020 | (-0.004, 0.045) | 0.105 |
| Political interest | -0.019 | (-0.089, 0.052) | 0.607 | -0.045 | (-0.104, 0.014) | 0.138 | 0.040*** | (0.024, 0.057) | 0.000 |
| Climate Scepticism | -0.160*** | (-0.202, -0.117) | 0.000 | -0.087*** | (-0.122, -0.051) | 0.000 | 0.025*** | (0.015, 0.035) | 0.000 |
| Quality of Participants | - | - | - | - | - | - | -0.006 | (-0.022, 0.010) | 0.454 |
| Recommendations | - | - | - | - | - | - | 0.062*** | (0.049, 0.076) | 0.000 |

****p* < .001;***p* < .05****p* < .05.

#### Mediation analysis

Mediation analysis was performed based on the counter-factual framework and the interventional effect (Vansteelandt and Daniel, 2017; Chan and Leung, 2020). The analysis was conducted in R using the intmed package (Chan and Leung, 2020) with 3000 simulations.

Results from the mediation analysis indicated that

- the indirect effect through Recommendations was statistically significant, p = 0.000; -63.575% of the total effect was mediated through Recommendations.
- the indirect effect through Quality of Participants was non-significant, p = 0.476; 1.462% of the total effect was mediated through Quality of Participants.
- the indirect effect through the dependence between mediators was non-significant, p = 0.966.
- the indirect effect through the interaction between mediators was non-significant, p = 0.969.
- the direct effect was statistically significant, p = 0.000.
- the total effect was statistically significant, p = 0.049.

## Table 12E – Mediation analysis results (binding DMPs)

|  | Estimates | 95% CI | p-value |
| --- | --- | --- | --- |
| Indirect effect mediated through Recommendations | 0.048*** | (0.028, 0.070) | 0.000 |
| Indirect effect mediated through Quality of Participants | -0.001 | (-0.007, 0.003) | 0.476 |
| Indirect effect mediated through the dependence between mediators | 0.000 | (-0.004, 0.004) | 0.966 |
| Indirect effect mediated through the interaction between mediators | 0.000 | (0.000, 0.000) | 0.969 |
| Direct effect | -0.118*** | (-0.187, -0.049) | 0.000 |
| Total effect | -0.072* | (-0.141, 0.000) | 0.049 |
| Proportion of effect mediated through Recommendations | -0.636 |  |  |
| Proportion of effect mediated through Quality of Participants | 0.015 |  |  |

***p <=.001; ***p* <.01; **p*<.05

# **Appendix F – Actual knowledge of the KBR (instead of self-report): operationalization, distribution and replication**

*Descriptive statistics*

## Table 1F – Questions about the KBR knowledge and distribution – Wave 1 (pre)

| Question | Which of the following issues are discussed by people participating in this citizens' assembly? | | | | | | | | | | | |
| --- | --- | --- | --- | --- | --- | --- | --- | --- | --- | --- | --- | --- |
| Answers | **Climate protection** | Tax system reform | Immigration | | Welfare system reform | Urbanization, housing and population growth | Education system reform | Diversifying the economy | Unemployment | | Reform the healthcare system | Don't know |
| % | 62% | 3% | 3% | | 2% | 8% | 3% | 2% | 3% | | 1% | 13% |
| Question | How have the participants of this citizens' assembly been selected? | | | | | | | |  | |  |  |
| Answers | **randomly selected from the Luxembourg population** | popularly elected by Luxembourgish citizens | based on their level of knowledge about the topic | | Don't know |  | | | | | | |
| % | 65% | 4% | 4% | | 27% |  | | | | | | |
| Question | Do you know who has initially decided to create this citizens' assembly in Luxembourg? | | | | | | | | | | | |
| Answers | **The Prime Minister** | The Green Party within the Luxembourg government | All parties of the Luxembourg Government) | | All parties by consensus in the Luxembourg C. of Dep. | The Europe Union has invited all member states to organize such citizens' assemblies | Don't know |  | | | | |
| % | 22% | 4% | 15% | | 7% | 8% | 44% |  |  |  |  |  |
|  | Respondents with at least one correct answer | Respondents with no correct answer | |  | | | | | | | | |
|  | 84% (939) | 16% (180) | |  | | | | | |  | | |

Table 2F – Questions about the KBR knowledge and distribution – Wave 2 (post)

| Question | Which of the following issues are discussed by people participating in this citizens' assembly? | | | | | | | | | | | |
| --- | --- | --- | --- | --- | --- | --- | --- | --- | --- | --- | --- | --- |
| Answers | **Climate protection** | Tax system reform | Immigration | | Welfare system reform | Urbanization, housing and population growth | Education system reform | Diversifying the economy | Unemployment | | Reform the healthcare system | Don't know |
| % | 56% | 4% | 2% | | 2% | 3% | 5% | 1% | 3% | | 1% | 22% |
| Question | How have the participants of this citizens' assembly been selected? | | | | | | | |  | |  |  |
| Answers | **randomly selected from the Luxembourg population** | popularly elected by Luxembourgish citizens | based on their level of knowledge about the topic | | Don't know |  | | | | | | |
| % | 64% | 4% | 7% | | 25% |  | | | | | | |
| Question | Do you know who has initially decided to create this citizens' assembly in Luxembourg? | | | | | | | | | | | |
| Answers | **The Prime Minister** | The Green Party within the Luxembourg government | All parties of the Luxembourg Government) | | All parties by consensus in the Luxembourg C. of Dep. | The Europe Union has invited all member states to organize such citizens' assemblies | Don't know |  | | | | |
| % | 15% | 2% | 14% | | 11% | 11% | 46% |  |  |  |  |  |
|  | Respondents with at least one correct answer | Respondents with no correct answer | |  | | | | | | | | |
|  | 80% (1157) | 20% (288) | |  | | | | | |  | | |

**Replication of Figure 3**

#### Descriptive statistics

The table below shows the descriptive statistics of all analyses variables. The overall sample size is 1805. There were 19.78% cases with missing data. Complete case analysis was used for the subsequent mediation analysis.

## Table 3F – Descriptive statistics of continuous variables (DMP support)

| Variables | N | Mean | SD | Median | IQR |
| --- | --- | --- | --- | --- | --- |
|  | | | | | |
| Organization of other DMPs | 1448 | 0.716 | 0.226 | 0.750 | 0.000 |
| Recommendations | 1448 | 3.296 | 0.951 | 3.000 | 1.000 |
| Quality of participants | 1448 | 3.725 | 0.776 | 4.000 | 1.000 |
| Exposure to KBR | 1448 | 0.376 | 0.245 | 0.500 | 0.500 |
| Age | 1448 | 3.981 | 1.724 | 4.000 | 2.000 |
| Climate scepticism | 1448 | 2.350 | 1.115 | 2.000 | 1.000 |
| Political interest | 1448 | 2.052 | 0.805 | 2.000 | 0.000 |
|  | | | | | |

## Table 4F – Descriptive statistics of categorical variables (DMP support)

| Variables | Levels | N | % |
| --- | --- | --- | --- |
|  | | | |
| Gender | Men | 819 | 56.561 |
|  | Women | 629 | 43.439 |
|  |  | | |
| Education | Lower secondary education | 139 | 9.599 |
|  | Short-cycle tertiary education | 730 | 50.414 |
|  | Bachelor or more | 579 | 39.986 |
|  | | | |

#### Regression analysis

The table below shows the estimates from the key regression models for the mediation analysis.

- The outcome variable, Organization of other DMPs, was modelled with regression
- The mediator, Recommendations, was modelled with regression
- The mediator, Quality of participants, was modelled with regression

The mediator models indicated that the exposure variable, Exposure to KBR, was

- significantly associated with the mediator, Recommendations, b = 0.419, 95% CI = (0.214, 0.624).
- significantly associated with the mediator, Quality of participants, b = 0.395, 95% CI = (0.227, 0.563).

After accounting for the effect of the mediators , the outcome model indicated that the treatment variable, Exposure to KBR, was significantly associated with Organization of other DMPs, b = 0.050, 95% CI = (0.003, 0.097).

The outcome model also indicated that

- Recommendations is significantly associated with the outcome, Organization of other DMPs, b = 0.073, 95% CI = (0.061, 0.085).
- Quality of participants is significantly associated with the outcome, Organization of other DMPs, b = 0.033, 95% CI = (0.019, 0.048).

The effect of Gender, Age, Education, Climate scepticism and Political interest were adjusted for in the above analyses.

## Table 5F – Results from key regression analyses (DMP support)

|  | Recommendations | | | Quality of participants | | | Organization of other DMPs | | |
| --- | --- | --- | --- | --- | --- | --- | --- | --- | --- |
| Variables | b | 95% CI | p-value | b | 95% CI | p-value | b | 95% CI | p-value |
| (Intercept) | 3.900*** | (3.593, 4.208) | 0.000 | 3.895*** | (3.643, 4.147) | 0.000 | 0.371*** | (0.277, 0.465) | 0.000 |
| Age | -0.070*** | (-0.100, -0.041) | 0.000 | 0.021 | (-0.003, 0.045) | 0.090 | -0.003 | (-0.010, 0.004) | 0.347 |
| Exposure to KBR | 0.419*** | (0.214, 0.624) | 0.000 | 0.395*** | (0.227, 0.563) | 0.000 | 0.050* | (0.003, 0.097) | 0.035 |
| Education: Bachelor or more (ref. Lower secondary) | -0.170 | (-0.344, 0.005) | 0.057 | -0.063 | (-0.207, 0.080) | 0.388 | -0.004 | (-0.044, 0.035) | 0.838 |
| Education: Short-cycle tert. Edu. (ref. Lower secondary) | -0.140 | (-0.308, 0.028) | 0.103 | -0.022 | (-0.160, 0.116) | 0.756 | 0.029 | (-0.009, 0.067) | 0.139 |
| Gender: Women ref. Men | 0.085 | (-0.015, 0.186) | 0.096 | 0.037 | (-0.045, 0.120) | 0.374 | 0.024* | (0.001, 0.046) | 0.041 |
| Political interest | -0.008 | (-0.073, 0.057) | 0.800 | -0.042 | (-0.095, 0.011) | 0.123 | -0.011 | (-0.025, 0.004) | 0.158 |
| Climate scepticism | -0.155*** | (-0.199, -0.111) | 0.000 | -0.126*** | (-0.163, -0.090) | 0.000 | -0.011* | (-0.022, -0.001) | 0.030 |
| Quality of participants | - | - | - | - | - | - | 0.033*** | (0.019, 0.048) | 0.000 |
| Recommendations | - | - | - | - | - | - | 0.073*** | (0.061, 0.085) | 0.000 |

***p < .001;**p < .05***p < .05.

#### Mediation analysis

Mediation analysis was performed based on the counter-factual framework and the interventional effect (Vansteelandt and Daniel, 2017; Chan and Leung, 2020). The analysis was conducted in R using the intmed package (Chan and Leung, 2020) with 3000 simulations.

Results from the mediation analysis indicated that

- the indirect effect through Recommendations was statistically significant, p = 0.000; 32.337% of the total effect was mediated through Recommendations.
- the indirect effect through Quality of participants was statistically significant, p = 0.000; 13.811% of the total effect was mediated through Quality of participants.
- the indirect effect through the dependence between mediators was non-significant, p = 0.998.
- the indirect effect through the interaction between mediators was non-significant, p = 0.999.
- the direct effect was statistically significant, p = 0.029.
- the total effect was statistically significant, p = 0.000.

## Table 6F – Mediation analysis results (DMP support)

|  | Estimates | 95% CI | p-value |
| --- | --- | --- | --- |
| Indirect effect mediated through Recommendations | 0.030*** | (0.016, 0.047) | 0.000 |
| Indirect effect mediated through Quality of participants | 0.013*** | (0.006, 0.022) | 0.000 |
| Indirect effect mediated through the dependence between mediators | 0.000 | (-0.008, 0.008) | 0.998 |
| Indirect effect mediated through the interaction between mediators | 0.000 | (0.000, 0.000) | 0.999 |
| Direct effect | 0.050* | (0.005, 0.097) | 0.029 |
| Total effect | 0.094*** | (0.046, 0.144) | 0.000 |
| Proportion of effect mediated through Recommendations | 0.323 |  |  |
| Proportion of effect mediated through Quality of participants | 0.138 |  |  |

****p* < .001;***p* < .05****p* < .05.

**Replication of Figure 4**

#### Descriptive statistics

The table below shows the descriptive statistics of all analyses variables. The overall sample size is 1805. There were 21.44% cases with missing data. Complete case analysis was used for the subsequent mediation analysis.

## Table 7F – Descriptive statistics of continuous variables (DMP participation)

| Variables | N | Mean | SD | Median | IQR |
| --- | --- | --- | --- | --- | --- |
|  | | | | | |
| Willingness to participate | 1418 | 0.550 | 0.311 | 0.600 | 0.500 |
| Recommendations | 1418 | 3.298 | 0.945 | 3.000 | 1.000 |
| Quality of participants | 1418 | 3.730 | 0.769 | 4.000 | 1.000 |
| Exposure to KBR | 1418 | 0.376 | 0.246 | 0.500 | 0.500 |
| Age | 1418 | 3.973 | 1.734 | 4.000 | 2.000 |
| Climate scepticism | 1418 | 2.441 | 1.221 | 2.000 | 2.000 |
| Political interest | 1418 | 2.055 | 0.807 | 2.000 | 1.000 |
|  | | | | | |

## Table 8F – Descriptive statistics of categorical variables (DMP participation)

| Variables | Levels | N | % |
| --- | --- | --- | --- |
|  | | | |
| Gender | Men | 801 | 56.488 |
|  | Women | 617 | 43.512 |
|  |  | | |
| Education | Lower secondary education | 135 | 9.520 |
|  | Short-cycle tertiary education | 713 | 50.282 |
|  | Bachelor or more | 570 | 40.197 |
|  | | | |

#### Regression analysis

The table below shows the estimates from the key regression models for the mediation analysis.

- The outcome variable, Willingness to participate, was modelled with regression
- The mediator, Recommendations, was modelled with regression
- The mediator, Quality of participants, was modelled with regression

The mediator models indicated that the exposure variable, Exposure to KBR, was

- significantly associated with the mediator, Recommendations, b = 0.477, 95% CI = (0.274, 0.680).
- significantly associated with the mediator, Quality of participants, b = 0.451, 95% CI = (0.282, 0.619).

After accounting for the effect of the mediators , the outcome model indicated that the treatment variable, Exposure to KBR, was significantly associated with Willingness to participate, b = 0.123, 95% CI = (0.059, 0.186).

The outcome model also indicated that

- Recommendations is significantly associated with the outcome, Willingness to participate, b = 0.050, 95% CI = (0.033, 0.067).
- Quality of participants is not significantly associated with the outcome, Willingness to participate, b = 0.017, 95% CI = (-0.003, 0.038).

The effect of Gender, Age, Education, Climate scepticism and Political interest were adjusted for in the above analyses.

## Table 9F – Results from key regression analyses (DMP participation)

|  | Recommendations | | | Quality of participants | | | Willingness to participate | | |
| --- | --- | --- | --- | --- | --- | --- | --- | --- | --- |
| Variables | b | 95% CI | p-value | b | 95% CI | p-value | b | 95% CI | p-value |
| (Intercept) | 3.839*** | (3.535, 4.143) | 0.000 | 3.778*** | (3.526, 4.030) | 0.000 | 0.529*** | (0.402, 0.656) | 0.000 |
| Age | -0.059*** | (-0.088, -0.029) | 0.000 | 0.019 | (-0.006, 0.044) | 0.130 | -0.025*** | (-0.035, -0.016) | 0.000 |
| Exposure to KBR | 0.477*** | (0.274, 0.680) | 0.000 | 0.451*** | (0.282, 0.619) | 0.000 | 0.123*** | (0.059, 0.186) | 0.000 |
| Education: Bachelor or more (ref. Lower secondary) | -0.200* | (-0.376, -0.023) | 0.026 | -0.051 | (-0.197, 0.095) | 0.495 | 0.143*** | (0.088, 0.198) | 0.000 |
| Education: Short-cycle tert. Edu. (ref. Lower secondary) | -0.152 | (-0.321, 0.016) | 0.077 | -0.023 | (-0.162, 0.117) | 0.751 | 0.053* | (0.001, 0.105) | 0.046 |
| Gender: Women ref. Men | 0.110* | (0.010, 0.209) | 0.031 | 0.051 | (-0.032, 0.133) | 0.231 | -0.028 | (-0.059, 0.003) | 0.079 |
| Political interest | 0.005 | (-0.060, 0.070) | 0.871 | -0.046 | (-0.100, 0.008) | 0.095 | -0.103*** | (-0.123, -0.083) | 0.000 |
| Climate scepticism | -0.160*** | (-0.200, -0.119) | 0.000 | -0.077*** | (-0.111, -0.044) | 0.000 | -0.006 | (-0.019, 0.007) | 0.370 |
| Quality of participants | - | - | - | - | - | - | 0.017 | (-0.003, 0.038) | 0.091 |
| Recommendations | - | - | - | - | - | - | 0.050*** | (0.033, 0.067) | 0.000 |

***p <=.001;***p* <.01;**p*

Mediation analysis

Mediation analysis was performed based on the counter-factual framework and the interventional effect (Vansteelandt and Daniel, 2017; Chan and Leung, 2020). The analysis was conducted in R using the intmed package (Chan and Leung, 2020) with 3000 simulations.

Results from the mediation analysis indicated that

- the indirect effect through Recommendations was statistically significant, p = 0.000; 15.075% of the total effect was mediated through Recommendations.
- the indirect effect through Quality of participants was non-significant, p = 0.086; 4.913% of the total effect was mediated through Quality of participants.
- the indirect effect through the dependence between mediators was non-significant, p = 0.957.
- the indirect effect through the interaction between mediators was non-significant, p = 0.962.
- the direct effect was statistically significant, p = 0.000.
- the total effect was statistically significant, p = 0.000.

## Table 10F – Mediation analysis results (DMP participation)

|  | Estimates | 95% CI | p-value |
| --- | --- | --- | --- |
| Indirect effect mediated through Recommendations | 0.024*** | (0.012, 0.039) | 0.000 |
| Indirect effect mediated through Quality of participants | 0.008 | (-0.001, 0.018) | 0.086 |
| Indirect effect mediated through the dependence between mediators | 0.000 | (-0.005, 0.005) | 0.957 |
| Indirect effect mediated through the interaction between mediators | 0.000 | (0.000, 0.000) | 0.962 |
| Direct effect | 0.124*** | (0.060, 0.186) | 0.000 |
| Total effect | 0.155*** | (0.092, 0.219) | 0.000 |
| Proportion of effect mediated through Recommendations | 0.151 |  |  |
| Proportion of effect mediated through Quality of participants | 0.049 |  |  |

****p* < .001;***p* < .05****p* < .05.

**Replication of Figure 5**

#### Descriptive statistics

The table below shows the descriptive statistics of all analyses variables. The overall sample size is 1805. There were 18.84% cases with missing data. Complete case analysis was used for the subsequent mediation analysis.

## Table 11F – Descriptive statistics of continuous variables (binding DMPs)

| Variables | N | Mean | SD | Median | IQR |
| --- | --- | --- | --- | --- | --- |
|  | | | | | |
| Binding DMPs | 1465 | 0.385 | 0.227 | 0.364 | 0.364 |
| Recommendations | 1465 | 3.291 | 0.949 | 3.000 | 1.000 |
| Quality of participants | 1465 | 3.720 | 0.778 | 4.000 | 1.000 |
| Exposure to KBR | 1465 | 0.374 | 0.245 | 0.500 | 0.500 |
| Age | 1465 | 3.977 | 1.734 | 4.000 | 2.000 |
| Climate scepticism | 1465 | 2.451 | 1.227 | 2.000 | 2.000 |
| Political interest | 1465 | 2.057 | 0.807 | 2.000 | 1.000 |
|  | | | | | |

## Table 12F – D escriptive statistics of categorical variables (binding DMPs)

| Variables | Levels | N | % |
| --- | --- | --- | --- |
|  | | | |
| Gender | Men | 829 | 56.587 |
|  | Women | 636 | 43.413 |
|  |  | | |
| Education | Lower secondary education | 141 | 9.625 |
|  | Short-cycle tertiary education | 739 | 50.444 |
|  | Bachelor or more | 585 | 39.932 |
|  | | | |

#### Regression analysis

The table below shows the estimates from the key regression models for the mediation analysis.

- The outcome variable, Binding DMPs, was modelled with regression
- The mediator, Recommendations, was modelled with regression
- The mediator, Quality of participants, was modelled with regression

The mediator models indicated that the exposure variable, Exposure to KBR, was

- significantly associated with the mediator, Recommendations, b = 0.443, 95% CI = (0.241, 0.645).
- significantly associated with the mediator, Quality of participants, b = 0.427, 95% CI = (0.259, 0.596).

After accounting for the effect of the mediators , the outcome model indicated that the treatment variable, Exposure to KBR, was significantly associated with Binding DMPs, b = -0.107, 95% CI = (-0.154, -0.059).

The outcome model also indicated that

- Recommendations is significantly associated with the outcome, Binding DMPs, b = 0.060, 95% CI = (0.047, 0.072).
- Quality of participants is not significantly associated with the outcome, Binding DMPs, b = 0.003, 95% CI = (-0.012, 0.018).

The effect of Gender, Age, Education, Climate scepticism and Political interest were adjusted for in the above analyses.

## Table 13F – Results from key regression analyses (binding DMPs)

|  | Recommendations | | | Quality of participants | | | Binding DMPs | | |
| --- | --- | --- | --- | --- | --- | --- | --- | --- | --- |
| Variables | b | 95% CI | p-value | b | 95% CI | p-value | b | 95% CI | p-value |
| (Intercept) | 3.871*** | (3.570, 4.171) | 0.000 | 3.767*** | (3.517, 4.017) | 0.000 | 0.121* | (0.027, 0.216) | 0.012 |
| Age | -0.059*** | (-0.089, -0.030) | 0.000 | 0.024 | (0.000, 0.048) | 0.055 | -0.012** | (-0.019, -0.005) | 0.001 |
| Exposure to KBR | 0.443*** | (0.241, 0.645) | 0.000 | 0.427*** | (0.259, 0.596) | 0.000 | -0.107*** | (-0.154, -0.059) | 0.000 |
| Education: Bachelor or more (ref. Lower secondary) | -0.211* | (-0.384, -0.037) | 0.017 | -0.064 | (-0.209, 0.080) | 0.383 | 0.015 | (-0.025, 0.056) | 0.457 |
| Education: Short-cycle tert. Edu. (ref. Lower secondary) | -0.166 | (-0.332, 0.000) | 0.050 | -0.047 | (-0.185, 0.092) | 0.509 | 0.015 | (-0.023, 0.054) | 0.437 |
| Gender: Women ref. Men | 0.113* | (0.015, 0.212) | 0.025 | 0.065 | (-0.017, 0.147) | 0.119 | 0.018 | (-0.005, 0.041) | 0.132 |
| Political interest | -0.002 | (-0.066, 0.062) | 0.955 | -0.040 | (-0.093, 0.014) | 0.146 | 0.029*** | (0.014, 0.044) | 0.000 |
| Climate scepticism | -0.158*** | (-0.198, -0.118) | 0.000 | -0.081*** | (-0.115, -0.048) | 0.000 | 0.026*** | (0.017, 0.036) | 0.000 |
| Quality of participants | - | - | - | - | - | - | 0.003 | (-0.012, 0.018) | 0.738 |
| Recommendations | - | - | - | - | - | - | 0.060*** | (0.047, 0.072) | 0.000 |

****p* < .001;***p* < .05****p* < .05.

#### Mediation analysis

Mediation analysis was performed based on the counter-factual framework and the interventional effect (Vansteelandt and Daniel, 2017; Chan and Leung, 2020). The analysis was conducted in R using the intmed package (Chan and Leung, 2020) with 3000 simulations.

Results from the mediation analysis indicated that

- the indirect effect through Recommendations was statistically significant, p = 0.000; -33.140% of the total effect was mediated through Recommendations.
- the indirect effect through Quality of participants was non-significant, p = 0.746; -1.265% of the total effect was mediated through Quality of participants.
- the indirect effect through the dependence between mediators was non-significant, p = 0.986.
- the indirect effect through the interaction between mediators was non-significant, p = 0.979.
- the direct effect was statistically significant, p = 0.000.
- the total effect was statistically significant, p = 0.000.

## Table 14F – Mediation analysis results (binding DMPs)

|  | Estimates | 95% CI | p-value |
| --- | --- | --- | --- |
| Indirect effect mediated through Recommendations | 0.026*** | (0.013, 0.041) | 0.000 |
| Indirect effect mediated through Quality of participants | 0.001 | (-0.005, 0.008) | 0.746 |
| Indirect effect mediated through the dependence between mediators | 0.000 | (-0.002, 0.002) | 0.986 |
| Indirect effect mediated through the interaction between mediators | 0.000 | (0.000, 0.000) | 0.979 |
| Direct effect | -0.106*** | (-0.154, -0.058) | 0.000 |
| Total effect | -0.079*** | (-0.127, -0.029) | 0.000 |
| Proportion of effect mediated through Recommendations | -0.331 |  |  |
| Proportion of effect mediated through Quality of participants | -0.013 |  |  |

****p* < .001;***p* < .05****p* < .05.

# **Appendix G – Causal mediation analysis for Figure 3, Figure 4 and Figure 5: full model specification**

**Figure 3: causal mediation analysis for DMP support**

#### Descriptive statistics

The table below shows the descriptive statistics of all analyses variables. The overall sample size is 1841. There were 19.61% cases with missing data. Complete case analysis wass used for the subsequent mediation analysis.

Table 1G – Descriptive statistics of continuous variables (DMP support)

| Variables | N | Mean | SD | Median | IQR |
| --- | --- | --- | --- | --- | --- |
|  | | | | | |
| Organization of other DMPs | 1480 | 0.714 | 0.226 | 0.750 | 0.250 |
| Recommendations | 1480 | 3.290 | 0.948 | 3.000 | 1.000 |
| Quality of participants | 1480 | 3.719 | 0.781 | 4.000 | 1.000 |
| Exposure to KBR | 1480 | 0.438 | 0.201 | 0.500 | 0.000 |
| Age | 1480 | 3.988 | 1.731 | 4.000 | 2.000 |
| Climate scepticism | 1480 | 2.355 | 1.116 | 2.000 | 1.000 |
| Political interest | 1480 | 2.050 | 0.802 | 2.000 | 0.000 |
|  | | | | | |

## Table 2G – Descriptive statistics of categorical variables (DMP support)

| Variables | Levels | N | % |
| --- | --- | --- | --- |
|  | | | |
| Gender | Men | 842 | 56.892 |
|  | Women | 638 | 43.108 |
|  |  | | |
| Education | Lower secondary education | 144 | 9.730 |
|  | Short-cycle tertiary education | 741 | 50.068 |
|  | Bachelor or more | 595 | 40.203 |
|  | | | |

#### Regression analysis

The table below shows the estimates from the key regression models for the mediation analysis.

- The outcome variable, Organization of other DMPs, was modelled with regression
- The mediator, Recommendations, was modelled with regression
- The mediator, Quality of Participants, was modelled with regression

The mediator models indicated that the exposure variable, Exposure to KBR, was

- significantly associated with the mediator, Recommendations, b = 0.380, 95% CI = (0.136, 0.624).
- significantly associated with the mediator, Quality of participants, b = 0.261, 95% CI = (0.059, 0.464).

After accounting for the effect of the mediators , the outcome model indicated that the treatment variable, Exposure to KBR, was significantly associated with Organization of other DMPs, b = 0.064, 95% CI = (0.008, 0.120).

The outcome model also indicated that

- Recommendations is significantly associated with the outcome, Organization of other DMPs, b = 0.072, 95% CI = (0.060, 0.084).
- Quality of participants is significantly associated with the outcome, Organization of other DMPs, b = 0.032, 95% CI = (0.018, 0.047).

The effect of Gender, Age, Education and Climate scepticism and political interest were adjusted for in the above analyses.

Table 3G – Results from key regression analyses (DMP support)

|  | Recommendations | | | Quality of participants | | | Organization of other DMPs | | |
| --- | --- | --- | --- | --- | --- | --- | --- | --- | --- |
| Variables | b | 95% CI | p-value | b | 95% CI | p-value | b | 95% CI | p-value |
| (Intercept) | 3.940*** | (3.628, 4.252) | 0.000 | 3.984*** | (3.724, 4.243) | 0.000 | 0.361*** | (0.265, 0.457) | 0.000 |
| Age | -0.074*** | (-0.103, -0.045) | 0.000 | 0.020 | (-0.004, 0.045) | 0.099 | -0.002 | (-0.009, 0.004) | 0.469 |
| Exposure to KBR | 0.380** | (0.136, 0.624) | 0.002 | 0.261* | (0.059, 0.464) | 0.012 | 0.064* | (0.008, 0.120) | 0.024 |
| Education: Bachelor or more (ref. Lower secondary) | -0.172* | (-0.343, -0.001) | 0.049 | -0.062 | (-0.204, 0.081) | 0.396 | -0.003 | (-0.042, 0.036) | 0.864 |
| Education: Short-cycle tert. Edu. (ref. Lower secondary) | -0.142 | (-0.307, 0.023) | 0.092 | -0.022 | (-0.159, 0.115) | 0.750 | 0.031 | (-0.007, 0.068) | 0.109 |
| Gender: Women ref. Men | 0.076 | (-0.023, 0.175) | 0.130 | 0.036 | (-0.046, 0.118) | 0.393 | 0.028* | (0.006, 0.051) | 0.014 |
| Climate scepticism | -0.014 | (-0.079, 0.050) | 0.660 | -0.056* | (-0.109, -0.003) | 0.040 | -0.013 | (-0.028, 0.002) | 0.083 |
| Political interest | -0.163*** | (-0.206, -0.119) | 0.000 | -0.138*** | (-0.174, -0.102) | 0.000 | -0.009 | (-0.020, 0.001) | 0.067 |
| Quality of participants | - | - | - | - | - | - | 0.032*** | (0.018, 0.047) | 0.000 |
| Recommendations | - | - | - | - | - | - | 0.072*** | (0.060, 0.084) | 0.000 |

***p <=.001;***p* <.01;**p* <.05.

#### Mediation analysis

Mediation analysis was performed based on the counter-factual framework and the interventional effect (Vansteelandt and Daniel, 2017; Chan and Leung, 2020). The analysis was conducted in R using the intmed package (Chan and Leung, 2020) with 3000 simulations.

Results from the mediation analysis indicated that

- the indirect effect through Recommendations was statistically significant, p = 0.003; 27.387% of the total effect was mediated through Recommendations.
- the indirect effect through Quality of participants was statistically significant, p = 0.010; 8.499% of the total effect was mediated through Quality of participants.
- the indirect effect through the dependence between mediators was non-significant, p = 0.994.
- the indirect effect through the interaction between mediators was non-significant, p = 0.993.
- the direct effect was statistically significant, p = 0.021.
- the total effect was statistically significant, p = 0.001.

## Table 4G – Mediation analysis results (DMP support)

|  | Estimates | 95% CI | p-value |
| --- | --- | --- | --- |
| Indirect effect mediated through Recommendations | 0.027** | (0.010, 0.046) | 0.003 |
| Indirect effect mediated through Quality of participants | 0.009* | (0.002, 0.018) | 0.010 |
| Indirect effect mediated through the dependence between mediators | 0.000 | (-0.010, 0.010) | 0.994 |
| Indirect effect mediated through the interaction between mediators | 0.000 | (0.000, 0.000) | 0.993 |
| Direct effect | 0.064* | (0.010, 0.119) | 0.021 |
| Total effect | 0.099*** | (0.041, 0.159) | 0.001 |
| Proportion of effect mediated through Recommendations | 0.274 |  |  |
| Proportion of effect mediated through Quality of participants | 0.085 |  |  |

****p* < .001;***p* < .05**p* < .05.

**Figure 4: causal mediation analysis (DMP participation)**

The table below shows the descriptive statistics of all analyses variables. The overall sample size is 1841. There were 21.35% cases with missing data. Complete case analysis was used for the subsequent mediation analysis.

Table 5G – Descriptive statistics of continuous variables (DMP participation)

| Variables | N | Mean | SD | Median | IQR |
| --- | --- | --- | --- | --- | --- |
|  | | | | | |
| Willingness to participate | 1448 | 0.549 | 0.310 | 0.600 | 0.500 |
| Recommendations | 1448 | 3.291 | 0.942 | 3.000 | 1.000 |
| Quality of participants | 1448 | 3.724 | 0.774 | 4.000 | 1.000 |
| Exposure to KBR | 1448 | 0.439 | 0.201 | 0.500 | 0.000 |
| Age | 1448 | 3.983 | 1.742 | 4.000 | 2.000 |
| Climate scepticism | 1448 | 2.449 | 1.225 | 2.000 | 2.000 |
| Political interest | 1448 | 2.052 | 0.804 | 2.000 | 0.000 |
|  | | | | | |

## Table 6G – Descriptive statistics of categorical variables (DMP participation)

| Variables | Levels | N | % |
| --- | --- | --- | --- |
|  | | | |
| Gender | Men | 822 | 56.768 |
|  | Women | 626 | 43.232 |
|  |  | | |
| Education | Lower secondary education | 139 | 9.599 |
|  | Short-cycle tertiary education | 723 | 49.931 |
|  | Bachelor or more | 586 | 40.470 |
|  | | | |

#### Regression analysis

The table below shows the estimates from the key regression models for the mediation analysis.

- The outcome variable, Willingness to participate, was modelled with regression
- The mediator, Recommendations, was modelled with regression
- The mediator, Quality of Participants, was modelled with regression

The mediator models indicated that the exposure variable, DMPknowledge4, was

- significantly associated with the mediator, Recommendations, b = 0.389, 95% CI = (0.143, 0.635).
- significantly associated with the mediator, Quality of Participants, b = 0.301, 95% CI = (0.095, 0.507).

After accounting for the effect of the mediators , the outcome model indicated that the treatment variable, DMPknowledge4, was significantly associated with Willingness to participate, b = 0.138, 95% CI = (0.062, 0.215).

The outcome model also indicated that

- Recommendations is significantly associated with the outcome, Willingness to participate, b = 0.050, 95% CI = (0.033, 0.067).
- Quality of Participants is not significantly associated with the outcome, Willingness to participate, b = 0.019, 95% CI = (0.000, 0.039).

The effect of Gender, Age, Education, Climate scepticism and Political interest were adjusted for in the above analyses.

## Table 7G – Results from key regression analyses (DMP participation)

|  | Recommendations | | | Quality of Participants | | | Willingness to participate | | |
| --- | --- | --- | --- | --- | --- | --- | --- | --- | --- |
| Variables | b | 95% CI | p-value | b | 95% CI | p-value | b | 95% CI | p-value |
| (Intercept) | 3.867*** | (3.555, 4.179) | 0.000 | 3.870*** | (3.609, 4.131) | 0.000 | 0.506*** | (0.377, 0.634) | 0.000 |
| Age | -0.063*** | (-0.092, -0.034) | 0.000 | 0.018 | (-0.007, 0.042) | 0.162 | -0.026*** | (-0.035, -0.017) | 0.000 |
| Exposure to KBR | 0.389** | (0.143, 0.635) | 0.002 | 0.301** | (0.095, 0.507) | 0.004 | 0.138*** | (0.062, 0.215) | 0.000 |
| Education: Bachelor or more (ref. Lower secondary) | -0.182* | (-0.356, -0.009) | 0.039 | -0.049 | (-0.194, 0.097) | 0.512 | 0.144*** | (0.091, 0.198) | 0.000 |
| Education: Short-cycle tert. Edu. (ref. Lower secondary) | -0.143 | (-0.309, 0.024) | 0.094 | -0.023 | (-0.162, 0.117) | 0.750 | 0.055* | (0.004, 0.106) | 0.036 |
| Gender: Women ref. Men | 0.109* | (0.010, 0.208) | 0.031 | 0.051 | (-0.032, 0.134) | 0.226 | -0.023 | (-0.053, 0.008) | 0.148 |
| Climate scepticism | -0.006 | (-0.071, 0.058) | 0.854 | -0.061* | (-0.115, -0.007) | 0.027 | -0.104*** | (-0.124, -0.084) | 0.000 |
| Political interest | -0.157*** | (-0.197, -0.118) | 0.000 | -0.087*** | (-0.121, -0.054) | 0.000 | -0.006 | (-0.018, 0.007) | 0.356 |
| Quality of participants | - | - | - | - | - | - | 0.019* | (0.000, 0.039) | 0.050 |
| Recommendations | - | - | - | - | - | - | 0.050*** | (0.033, 0.067) | 0.000 |

***p <=.001;***p* <.01;**p*<.05.

#### Mediation analysis

Mediation analysis was performed based on the counter-factual framework and the interventional effect (Vansteelandt and Daniel, 2017; Chan and Leung, 2020). The analysis was conducted in R using the intmed package (Chan and Leung, 2020) with 3000 simulations.

Results from the mediation analysis indicated that

- the indirect effect through Recommendations was statistically significant, p = 0.001; 11.762% of the total effect was mediated through Recommendations.
- the indirect effect through Quality of Participants was non-significant, p = 0.054; 3.226% of the total effect was mediated through Quality of Participants.
- the indirect effect through the dependence between mediators was non-significant, p = 0.983.
- the indirect effect through the interaction between mediators was non-significant, p = 0.961.
- the direct effect was statistically significant, p = 0.001.
- the total effect was statistically significant, p = 0.000.

## Table 8G – Mediation analysis results (DMP participation)

|  | Estimates | 95% CI | p-value |
| --- | --- | --- | --- |
| Indirect effect mediated through Recommendations | 0.020*** | (0.006, 0.035) | 0.001 |
| Indirect effect mediated through Quality of Participants | 0.006* | (0.000, 0.015) | 0.050 |
| Indirect effect mediated through the dependence between mediators | 0.000 | (-0.007, 0.007) | 0.983 |
| Indirect effect mediated through the interaction between mediators | 0.000 | (0.000, 0.000) | 0.961 |
| Direct effect | 0.137*** | (0.061, 0.215) | 0.001 |
| Total effect | 0.163*** | (0.086, 0.243) | 0.000 |
| Proportion of effect mediated through Recommendations | 0.118 |  |  |
| Proportion of effect mediated through Quality of Participants | 0.032 |  |  |

****p* < .001;***p* < .01;**p* < .05.

**Figure 5: causal mediation analysis (binding DMPs)**

#### Descriptive statistics

The table below shows the descriptive statistics of all analyses variables. The overall sample size is 1841. There were 18.69% cases with missing data. Complete case analysis was used for the subsequent mediation analysis.

Table 9G – Descriptive statistics of continuous variables (binding DMPs)

| Variables | N | Mean | SD | Median | IQR |
| --- | --- | --- | --- | --- | --- |
|  | | | | | |
| Binding DMPs | 1497 | 0.386 | 0.228 | 0.364 | 0.364 |
| Recommendations | 1497 | 3.285 | 0.946 | 3.000 | 1.000 |
| Quality of participants | 1497 | 3.714 | 0.782 | 4.000 | 1.000 |
| Exposure to KBR | 1497 | 0.438 | 0.200 | 0.500 | 0.000 |
| Age | 1497 | 3.983 | 1.741 | 4.000 | 2.000 |
| Climate scepticism | 1497 | 2.458 | 1.230 | 2.000 | 2.000 |
| Political interest | 1497 | 2.055 | 0.804 | 2.000 | 0.000 |
|  | | | | | |

## Table 10G – Descriptive statistics of categorical variables (binding DMPs)

| Variables | Levels | N | % |
| --- | --- | --- | --- |
|  | | | |
| Gender | Men | 852 | 56.914 |
|  | Women | 645 | 43.086 |
|  |  | | |
| Education | Lower secondary education | 146 | 9.753 |
|  | Short-cycle tertiary education | 750 | 50.100 |
|  | Bachelor or more | 601 | 40.147 |
|  | | | |

#### Regression analysis

The table below shows the estimates from the key regression models for the mediation analysis.

- The outcome variable, Binding DMPs, was modelled with regression
- The mediator, Recommendations, was modelled with regression
- The mediator, Quality of Participants, was modelled with regression

The mediator models indicated that the exposure variable, DMPknowledge4, was

- significantly associated with the mediator, Recommendations, b = 0.373, 95% CI = (0.129, 0.617).
- significantly associated with the mediator, Quality of Participants, b = 0.273, 95% CI = (0.067, 0.479).

After accounting for the effect of the mediators , the outcome model indicated that the treatment variable, DMPknowledge4, was significantly associated with Binding DMPs, b = -0.088, 95% CI = (-0.145, -0.030).

The outcome model also indicated that

- Recommendations is significantly associated with the outcome, Binding DMPs, b = 0.058, 95% CI = (0.045, 0.070).
- Quality of Participants is not significantly associated with the outcome, Binding DMPs, b = -0.004, 95% CI = (-0.019, 0.011).

The effect of Gender, Age, Education, Climate scepticism and Political interest were adjusted for in the above analyses.

## Table 11G – Results from key regression analyses (binding DMPs)

|  | Recommendations | | | Quality of Participants | | | Binding DMPs | | |
| --- | --- | --- | --- | --- | --- | --- | --- | --- | --- |
| Variables | b | 95% CI | p-value | b | 95% CI | p-value | b | 95% CI | p-value |
| (Intercept) | 3.899*** | (3.592, 4.206) | 0.000 | 3.858*** | (3.600, 4.117) | 0.000 | 0.145** | (0.048, 0.241) | 0.003 |
| Age | -0.065*** | (-0.093, -0.036) | 0.000 | 0.023 | (-0.001, 0.047) | 0.066 | -0.012*** | (-0.019, -0.005) | 0.001 |
| Exposure to KBR | 0.373** | (0.129, 0.617) | 0.003 | 0.273** | (0.067, 0.479) | 0.009 | -0.088** | (-0.145, -0.030) | 0.003 |
| Education: Bachelor or more (ref. Lower secondary) | -0.200* | (-0.370, -0.030) | 0.021 | -0.058 | (-0.201, 0.086) | 0.430 | 0.014 | (-0.026, 0.054) | 0.498 |
| Education: Short-cycle tert. Edu. (ref. Lower secondary) | -0.159 | (-0.322, 0.005) | 0.057 | -0.042 | (-0.179, 0.096) | 0.555 | 0.013 | (-0.025, 0.052) | 0.497 |
| Gender: Women ref. Men | 0.109* | (0.011, 0.206) | 0.029 | 0.067 | (-0.015, 0.149) | 0.109 | 0.016 | (-0.007, 0.039) | 0.180 |
| Climate scepticism | -0.013 | (-0.076, 0.051) | 0.696 | -0.057* | (-0.111, -0.003) | 0.038 | 0.035*** | (0.020, 0.050) | 0.000 |
| Political interest | -0.155*** | (-0.194, -0.116) | 0.000 | -0.091*** | (-0.124, -0.058) | 0.000 | 0.024*** | (0.015, 0.034) | 0.000 |
| Quality of participants | - | - | - | - | - | - | -0.004 | (-0.019, 0.011) | 0.609 |
| Recommendations | - | - | - | - | - | - | 0.058*** | (0.045, 0.070) | 0.000 |

***p <=.001; ***p* <.01; **p*<.05.

#### Mediation analysis

Mediation analysis was performed based on the counter-factual framework and the interventional effect (Vansteelandt and Daniel, 2017; Chan and Leung, 2020). The analysis was conducted in R using the intmed package (Chan and Leung, 2020) with 3000 simulations.

Results from the mediation analysis indicated that

- the indirect effect through Recommendations was statistically significant, p = 0.001; -30.663% of the total effect was mediated through Recommendations.
- the indirect effect through Quality of Participants was non-signifcant, p = 0.603; 1.314% of the total effect was mediated through Quality of Participants.
- the indirect effect through the dependence between mediators was non-signifcant, p = 0.997.
- the indirect effect through the interaction between mediators was non-signifcant, p = 0.994.
- the direct effect was statistically significant, p = 0.001.
- the total effect was statistically significant, p = 0.030.

## Table 12G – Mediation analysis results (binding DMPs)

|  | Estimates | 95% CI | p-value |
| --- | --- | --- | --- |
| Indirect effect mediated through Recommendations | 0.022** | (0.007, 0.038) | 0.001 |
| Indirect effect mediated through Quality of Participants | -0.001 | (-0.006, 0.003) | 0.603 |
| Indirect effect mediated through the dependence between mediators | 0.000 | (-0.003, 0.003) | 0.997 |
| Indirect effect mediated through the interaction between mediators | 0.000 | (0.000, 0.000) | 0.994 |
| Direct effect | -0.088** | (-0.146, -0.028) | 0.001 |
| Total effect | -0.068* | (-0.128, -0.007) | 0.030 |
| Proportion of effect mediated through Recommendations | -0.307 |  |  |
| Proportion of effect mediated through Quality of Participants | 0.013 |  |  |

****p* < .001;***p* < .01****p* < .05.
